# Coping with Ineffective Overlap in Multilocus Phylogenetics

**DOI:** 10.1093/sysbio/syaf044

**Published:** 2025-07-03

**Authors:** Ana Serra Silva, Karen Siu-Ting, Christopher J Creevey, Davide Pisani, Mark Wilkinson

**Affiliations:** Herpetology Lab, Science Group, The Natural History Museum, Cromwell Road, London SW7 5BD, UK; School of Earth Sciences, University of Bristol, Queens Road, Bristol BS8 1RL, UK; Centre for Life's Origins and Evolution, Department of Genetics, Evolution and Environment, University College London, Gower Street, London WC1E 6BT, UK; School of Biological Sciences, Queen’s University Belfast, Chlorine Gardens, Belfast BT7 1NN, UK; School of Biological Sciences, Queen’s University Belfast, Chlorine Gardens, Belfast BT7 1NN, UK; School of Earth Sciences, University of Bristol, Queens Road, Bristol BS8 1RL, UK; School of Biological Sciences, University of Bristol, Tyndall Avenue, Bristol BS8 1RL, UK; Herpetology Lab, Science Group, The Natural History Museum, Cromwell Road, London SW7 5BD, UK

**Keywords:** Ineffective overlap, matrix representation of splits, phylogenomics, targeted taxon/locus sampling, taxonomic instability, terraces

## Abstract

Missing data is a long-standing issue in phylogenetic inference, which often results in high levels of taxonomic instability, obscuring otherwise well-supported relationships. Multiple approaches have been developed to deal with the negative effects of ineffective overlap on tree resolution, often by identifying taxa for removal. Here, we repurpose a heuristic method developed to identify unstable taxa in morphological data matrices, concatabominations, and combine it with a novel gene-tree jackknifing on matrix representation of trees to identify candidates for targeted sequencing. Using a multilocus caecilian data set, we illustrate the method’s capacity to identify candidate taxa and loci for additional sequencing, compare the results with those of the mathematics-based gene sampling sufficiency approach, and explore the terrace space associated with the multilocus data set. We show that our approach yields tractable numbers of loci/taxa for targeted sequencing that successfully mitigate topological instability due to ineffective overlap, even when modest amounts of data are added.

Whether they be pursued through simultaneous analyses of concatenated gene sequences or through species-tree/supertree amalgamation of individual gene-trees, the success of phylogenomic studies is contingent upon which taxa are shared between data partitions. *Effective overlap* (Wilkinson and Cotton 2006), or *decisive taxon coverage* (Sanderson et al. 2010; Steel and Sanderson 2010; Steel 2016), is needed to avoid large numbers of equally optimal trees (terraces, Sanderson et al. 2011, 2015), and poorly resolved or supported consensus trees (Fig. 1). Given a tree *T* and a taxon coverage pattern (a set of leaf sets) *S, S* is decisive for *T* if *T* is the unique supertree of the subtrees of *T* induced by *S* (Sanderson et al. 2010; Steel and Sanderson 2010; Dobrin et al. 2018). For example, the taxon coverage in Figure 1a (*S* = {A,B,C} and {A,C,D}) is decisive for *T* = (((A,B),C),D) because the subtrees induced by *S*, ((A,B),C) and ((A,C),D), entail *T*. In contrast, taxon coverage in Figure 1b, (*S* = {A,B,C} and {A,B,D}), is not decisive because the subtrees of *T* induced by *S*, ((A,B),C) and ((A,B),D), do not entail *T*. Rather they permit the three possible supertrees (((A,B),C),D), ((A,B),(C,D)), and (((A,B),D),C), the consensus of which is incompletely resolved. A taxon coverage pattern may be decisive for any tree (e.g., when one marker is available for all the leaves), but otherwise the same taxon coverage pattern may be decisive for some tree *T* but not for another tree *T*, reflecting the importance of the underlying tree in determining whether a taxon coverage pattern is decisive or produces ineffective overlap. Taxon coverage decisiveness underpins the concept of phylogenetic terraces in multilocus data, where terraces are sets of *exactly* equally optimal trees such that each data partition (tree) induces identical subtrees (Sanderson et al. 2011).

**Figure 1.**
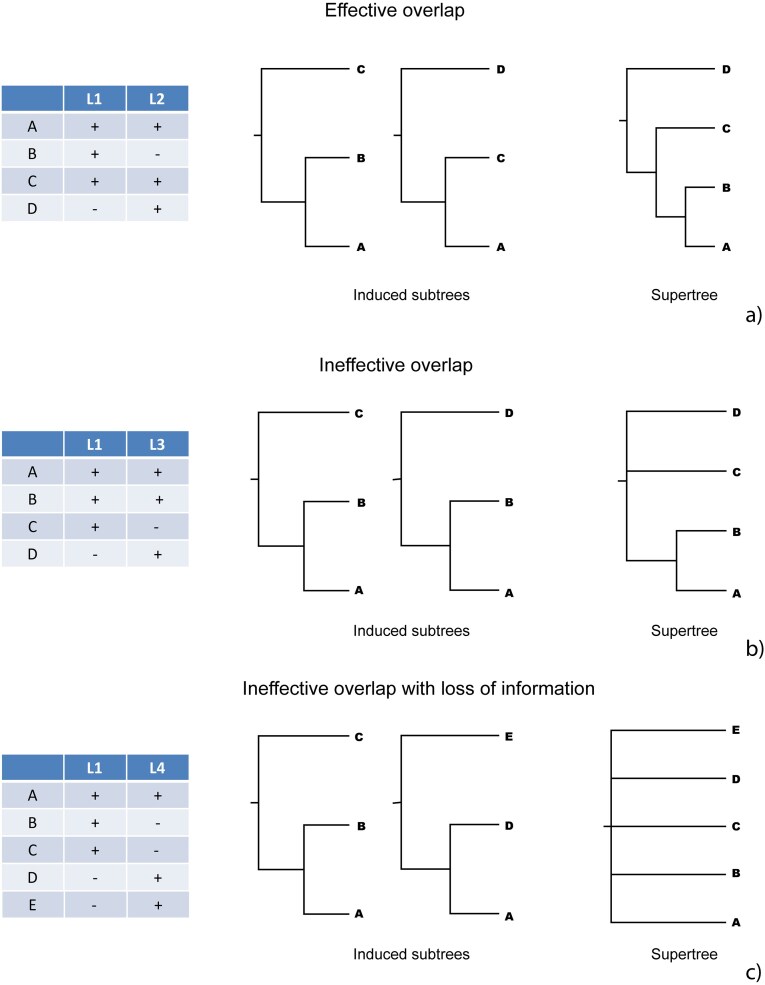
Patterns of overlap for three sets of subtrees (or data partitions in the supermatrix case). In panel (a), the pattern of overlap is effective (i.e., the taxon coverage is decisive) because the two subtrees induced encode a unique supertree, whereas in panel (b) the induced subtrees encode three unique supertrees, illustrating ineffective overlap (nondecisive taxon coverage). In panel (c), extreme ineffective overlap with complete loss of topological information is illustrated.

Although decisive taxonomic coverage reflects the effectiveness and not just the amount of overlap, some progress in understanding and diagnosing decisive taxon coverage in phylogenomic data has been made focusing only upon simple patterns of the co-occurrence (presence and absence) of genes and taxa (Sanderson et al. 2010; Steel and Sanderson 2010; Steel 2016). Nonetheless, taking into account additional phylogenetic information provided by the relationships supported by individual gene-trees might prove useful for diagnosing and developing strategies for ameliorating or overcoming ineffective overlap (Wilkinson and Cotton 2006).

Here, we view ineffective overlap as a missing data problem and investigate the potential of concatabominations, a technique developed for the *a priori* detection of taxa that are relatively unstable due to missing data (Siu-Ting et al. 2015), as a tool in multilocus phylogenetics. We explain how concatabominations can be applied to collections of gene-trees to diagnose ineffective overlap, introduce a gene-tree jackknifing approach to help identify candidate loci for targeted sequencing, and illustrate the approach by constructing multilocus supertrees for caecilian amphibians. We further discuss and compare the alternative approach based on patterns of co-occurrence of genes and taxa without consideration of the phylogenetic information in individual gene-trees using both the caecilian phylogeny and a selection of the data sets employed by Dobrin et al. (2018) to explore the prevalence of terraces in multilocus concatenated data matrices.

## Materials and Methods

### Application of Concatabominations to Multilocus Data

Concatabomination analysis was introduced by Siu-Ting et al. (2015) as a heuristic extension to safe taxonomic reduction (STR, Wilkinson 1995), an often-used *a priori* technique for identifying leaves (terminal taxa) that are relatively unstable (rogues) in phylogenetic analyses due to their patterns of missing data. Concatabominations are artificial constructs comprising the combined, non-overlapping data for a pair of leaves. Combining the data is analogous to forcing the leaves together on a tree. If such concatabominations add no homoplasy to the data (judged using character compatibility in the original version of the method), this suggests no cost to forcing the leaves together on a tree. Any leaf that participates in many such no cost concatabominations (and by analogy can go in many places on equally optimal trees) are taken as candidate unstable taxa.

**Figure 2.**
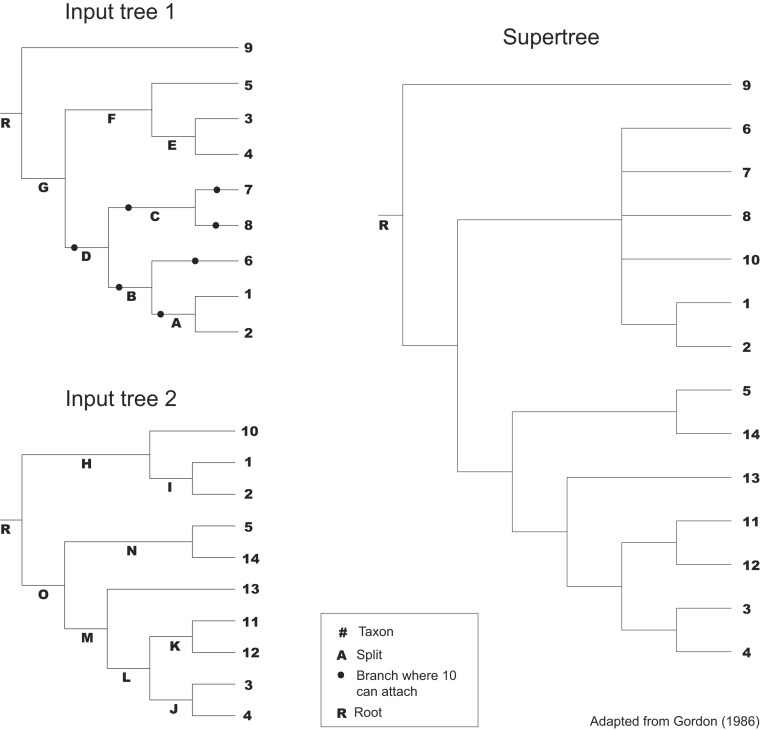
Gordon (1986) supertree.

Although originally developed for discrete morphological character data, STR has also been applied to matrix representations (MRs) of trees used in multilocus/phylogenomic supertree construction (e.g., Cardillo et al. 2004; Akanni et al. 2015). Similarly, rather than applying concatabominations directly to sequence data, we propose applying this approach to MRs of gene-trees in multilocus and phylogenomic studies. Figure 2 alongside Table 1 illustrates the principles of the approach and what may be gained from using it with a simple, much used and well-understood, hypothetical example due to Gordon (1986). In this example, there are two gene-trees (gene A and gene B, Fig. 2) with only a partially overlapping leaf set of five taxa (1–5). The two trees are compatible and there are seven plenary (i.e., including all the leaves) supertrees that display both of the gene-trees. The multiplicity of supertrees is a consequence of a degree of ineffective overlap (lack of decisive coverage) between the gene-trees. Specifically, the seven supertrees differ only in the placement of a single leaf (10), which behaves as a rogue taxon leading to poor resolution of one part of the strict consensus (SC, Sokal and Rohlf 1981) of the supertrees.

**Table 1.**
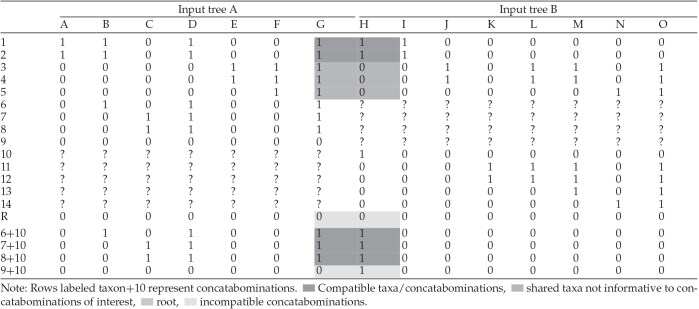
Concatabominations example. MR of Gordon’s (1986) input trees (Fig. 2), with rows representing taxa and columns representing splits.

Table 1 includes the standard MRs (Ragan 1992) of the two gene-trees of Figure 2 together with a subsample of the possible concatabominations of leaves (just those including leaf 10). Concatabominations of 10 with leaf 9 introduces a pairwise incompatibility into the MR indicating a cost to forcing leaves 9 and 10 together. In contrast, there is no incompatibility and no cost to forcing leaf 10 with leaves 6, 7, or 8 suggesting, as we already know, that there is ineffective overlap in the taxon sampling of the two genes resulting in rogue taxon behavior of leaf 10 with respect to leaves 6, 7, and 8. The concatabomination analysis supports two conclusions. First, it suggests that the rogue taxon behavior can be avoided by excluding either leaf 10 or leaves 6, 7, and 8 from the phylogenomic analyses. Clearly, the first option would, all other things being equal, be preferable. Second, it suggests that, all other things being equal, effective overlap could be achieved most simply and efficiently by obtaining sequence data of gene A for leaf 10.

In practice, the concatabominations pipeline outputs networks of potential taxonomic equivalence (concatabomination network hereafter) that can be visualized and manipulated in Cytoscape (Shannon et al. 2003), allowing users to explore how effective overlap changes as putative unstable (highly connected) taxa are removed, without the need for reanalyses of the modified matrices.

### Gene-Tree Jackknifing with Concatabominations

A more formal approach to identifying genes that might be targeted for additional sequencing so as to reduce ineffective overlap can be obtained by comparing the numbers of edges in the concatabominations graph with or without each of the gene-trees (i.e., through a first-order gene-tree jackknife). The greater the number of edges in the concatabominations graph, the greater the instability and ineffective overlap. If the removal of a gene-tree from the matrix increases the amount of instability recovered by the concatabominations pipeline (i.e., number of edges), then that gene-tree is considered “stabilizing,” and the locus is a candidate for a targeted increase in taxonomic sampling. For ease of interpretation, self-loops (edges connecting a taxon to itself) were removed from all graphs used in this study. A simplified version of the algorithm and a nonempirical example are provided in the Supplementary Material.

### Gymnophiona: Caecilian Amphibians

Gymnophiona Müller, 1832 is the least speciose of the three extant amphibian orders, with ca. 220 currently recognized species in 10 families. Caecilians are found primarily in tropical and subtropical environments, with most species being fossorial as adults and are easily distinguished from frogs and salamanders due to their elongated, annulated, and limbless bodies (e.g., Wilkinson 2012). Other than the addition of the family Chikilidae Kamei, San Mauro, Gower, Van Bocxlaer, Sherratt, Thomas, Babu, Bossuyt, Wilkinson, and Biju, 2012 (Kamei et al. 2012) and the replacement of the family-group name Indotyphliidae Lescure, Renous, and Gasc, 1986 with Grandisoniidae Lescure, Renous, and Gasc, 1986, which has priority (Dubois et al. 2021), the family-level classification of caecilians has been stable since Wilkinson et al.’s (2011) taxonomic revision.

Because of their habits, caecilians are not as frequently encountered or collected as other more conspicuous herpetological components of tropical ecosystems (e.g., Wilkinson 2012). Consequently, many species are known from very few specimens (e.g., Wilkinson, 2020). Thus, caecilians are a taxon for which targeted taxon/locus sampling may be important, and have been previously used to explore an information-based approach (Goldman 1998; Goldman et al. 2000) that addresses which data (which genes for which taxa) might be added to best improve unresolved or poorly supported relationships (San Mauro et al. 2009, 2012).

#### Data collection and processing

All caecilian nucleotide data available in GenBank (Benson et al. 2008) on 14 March 2022 were downloaded. To filter synonyms from the downloaded data, the taxonomic information provided by GenBank was checked using the Amphibian Species of the World v.6.0 database (Frost 2024). Unless included in previous comprehensive phylogenetic analyses (e.g., San Mauro et al. 2014), sequences identified to the species or genus level that included *aff., cf*., or *sp*. in their updated taxonomy were discarded. All loci with data for fewer than three caecilian species were discarded (e.g., tyrosinase), as was rhodopsin due to alignment quality. This resulted in a data set of 94 caecilian taxa, of which 88 are currently recognized species (89 with the duplicate added due to suspected non-monophyly, see later), 1 is identified only to genus and 4 are candidate species (*cf*.), and 24 loci: 15 mitochondrial and 9 nuclear loci (Table 2).

**Table 2.** Sampled loci, best-fit models, and alignment details, including length in base pairs and taxonomic coverage.

Locus	Number of taxa	Taxonomic coverage	Alignment length	Best-fit model
**mtDNA**				
12S rRNA	84	0.80	757	GTR+I+G
16S rRNA pre-jackknife	99	0.94	1229	GTR+I+G
16S rRNA post-jackknife	100	0.95	1142	GTR+I+G
ATP6	53	0.50	684	GTR+I+G
ATP8	53	0.50	157	GTR+I+G
COX1	86	0.82	1540	GTR+I+G
COX2	53	0.50	688	GTR+I+G
COX3	53	0.50	784	GTR+I+G
CYTB	81	0.77	1141	GTR+I+G
ND1	54	0.51	963	GTR+I+G
ND2	55	0.52	1035	GTR+I+G
ND3	53	0.50	343	GTR+I+G
ND4	53	0.50	1368	GTR+I+G
ND4L	53	0.50	297	GTR+I+G
ND5	53	0.50	1781	GTR+I+G
ND6	53	0.50	492	GTR+I+G
**nucDNA**				
18S rRNA	10	0.09	1775	GTR+G
28S rRNA	19	0.18	697	GTR+I+G
BDNF	13	0.12	713	GTR+I+G
CXCR4	33	0.31	644	HKY+I+G
H3A	25	0.24	328	GTR+G
NCX1	34	0.32	1304	GTR+I+G
RAG1	65	0.62	1509	GTR+I+G
SIA	13	0.12	432	GTR+I
SLC8A3	32	0.30	1121	GTR+I+G

Note: Loci are divided between mitochondrial (mtDNA) and nuclear (nucDNA).

From the filtered sequence data, only one sequence was selected per species per locus, except for *Gymnopis multiplicata* Peters, 1874, because the group of individuals that have been assigned to this species have been inferred to be paraphyletic with respect to *Dermophis mexicanus* (Duméril and Bibron, 1841) (San Mauro et al. 2014). The criteria for within species sequence selection were, in order (i) one voucher specimen–multiple loci, (ii) sequence length, (iii) most recent sequence, and (iv) valid taxon (not synonym) in sequence description.

Data were also downloaded for the following outgroups: the sarcopterigians *Latimeria chalumnae* Smith, 1939 and *Protopterus annectens* (Owen, 1839); the amniotes *Mus musculus* Linnaeus, 1758, *Anolis carolinensis* Voigt, 1832 and *Gallus gallus* (Linnaeus, 1758); the caudates *Lyciasalamandra atifi* (Baolu, 1967), *Andrias davidianus* (Blanchard, 1871) and *Ranodon sibiricus* Kessler, 1866; and the anurans *Leiopelma archeyi* Turbott, 1942, *Bombina orientalis* (Boulenger, 1890) and *Xenopus laevis* (Daudin, 1802). These outgroups were chosen because they are model organisms and thus likely to have sequence data for all/most sampled loci and/or due to their inclusion in prior systematic studies (San Mauro et al. 2014; Siu-Ting et al. 2019). For GenBank accession numbers, see Table S1 for nuclear loci (nucDNA) and Table S2 for mitogenomes and mitochondrial loci (mtDNA).

All loci were aligned using the MAFFT v.7.450 (Katoh and Standley 2013) plugin for Geneious Prime v.2022.0.2 (https://www.geneious.com, Kearse et al. 2012), under automatic algorithm selection. To remove hypervariable regions and trim ragged ends, all multiple sequence alignments were processed with Gblocks v.0.91b (Castresana 2002) under the settings: “minimum number of sequences for a flank position” = $\frac{n}{2} + 1$, where *n* is the total number of sequences in the alignment, “maximum number of contiguous nonconserved positions” = 10, “minimum length of a block” = 5, and “allowed gap positions” = “with half.”

#### Phylogenetic analyses

Nucleotide substitution models were fitted using jModelTest v.2.1.7 (Darriba et al. 2012), with the number of substitution models set to three (see Table 2 for selected models). Gene-trees were inferred with MrBayes v.3.2.7 (Ronquist et al. 2012), running two independent runs with four chains for 10 million generations, sampled every 1000 generations with a relative burn-in of 25%, and summarized with the majority-rule consensus (MRC, Margush and McMorris 1981). Run convergence was checked using an average standard deviation of split frequencies $\le$0.01, and a potential scale reduction factor of $\approx$1.00, if either measure was not met the analyses were run for an additional 10 million generations. Codon positions were not taken into account due to the use of Gblocks.

Supertrees were constructed with the standard MR with parsimony (MRP) approach (Baum and Ragan 2004). MRCs for inferred gene-trees were converted to their MRs and these concatenated using p4 v.1.2.0 (Foster 2004). Parsimony analyses were run in PAUP* v.4.a169 (Swofford 2003) with 1000 replicates of random addition and “MulTrees” selected, *Latimeria* Smith, 1939, set as the outgroup, and all other parameters unchanged from their defaults. The inferred trees were summarized with the SC. Given that the primary aim of this study is to illustrate and compare approaches of identifying and reducing ineffective overlap between loci rather than producing the best inference of caecilian phylogeny *per se*, MRP was used and MRC trees chosen over gene-trees with highest posterior probabilities to increase the chances of exemplifying ineffective overlap. Additional analyses with concatenated matrices, gene-trees inferred under maximum likelihood, and Astral-III (Zhang et al. 2018) supertrees are available in the Supplementary Material.

### Testing for Taxonomic Instability with Concatabominations and Gene-Tree Jackknifing

To test for ineffective overlap, the concatenated MR of the inferred gene-trees was run though the concatabominations pipeline (Siu-Ting et al. 2015), and visualized with Cytoscape v.3.7.1 (Shannon et al. 2003). To investigate which gene-trees are the best candidates for increased taxonomic sampling to mitigate ineffective overlap, the MR was subjected to a first-order gene-tree jackknife with each modified MR analyzed with the concatabominations pipeline.

Additionally, we applied concatabominations and gene-tree jackknifing to a selection of the data sets used by Dobrin et al. (2018) to explore the theoretical terrace space occupied by a variety of phylogenomic data sets (see Table 4). Individual loci were extracted from the concatenated matrices and all loci with fewer than four taxa (the minimum number required for an informative unrooted tree) were discarded as uninformative. To enhance comparison with Dobrin et al. (2018), gene-trees were inferred with RAxML v.8.2.12 (Stamatakis 2014), with the multiple tree search set to 1000 runs without bootstrap and with GTR+I+G as the substitution model for all gene-trees, and the best trees were used as input for the MRP analyses. The MRP searches were run under the same settings as the caecilian MRP analyses. In cases where these parameters lead to memory allocation errors, the analyses were re-run with steepest descent selected (“hsearch steepest=yes”).

**Table 3.** Concatabominations with tree jackknife results—number of edges excludes self-loops.

MR	Taxa in network	Edges in network	Taxa in jackknifed locus
All loci	105	59	NA
All loci, no *Hypogeophis montanus*	104	46	NA
**No 12S rRNA**	105	61 (27)	84
**No 16S rRNA**	103	135	99
No 18S rRNA	105	59 (29)	10
No 28S rRNA	105	59 (59)	19
No ATP6	105	59	53
No ATP8	105	59	53
No BDNF	104	25	13
**No COX1**	105	71 (27)	86
No COX2	105	59 (27)	53
No COX3	105	59	53
No CXCR4	105	59 (29)	33
**No CYTB**	104	63 (27)	81
**No H3A**	105	61 (59)	25
No NCX1	105	59 (29)	34
**No ND1**	105	63 (27)	54
No ND2	105	59 (27)	55
No ND3	105	59	53
No ND4	105	59	53
No ND4L	105	59	53
No ND5	105	59	53
No ND6	105	59	53
No RAG1	105	59 (27)	65
No SIA	105	59 (59)	13
No SLC8A3	105	59 (29)	32
With 16S rRNA for *H. montanus*	105	23	NA

Note: Candidate loci for targeted taxon sampling highlighted in bold text. Values in parentheses correspond to the number of edges present in “All loci” matrices with *H. montanus* pseudo-data added to the jackknifed locus.

**Table 4.** Results of the gene sampling sufficiency ($\zeta$) and terrace analyses on the selection of Dobrin et al.’s (2018) data sets, pre- and post-jackknife Gymnophiona data sets, and Gordon’s (1986) supertree (ST).

			Supertree					Comparison		Terraces			
Data set	Taxa	Loci	$\rho$	CIC	*d*	*k*_min_ *P* = 0.05	$\zeta$	$\Delta$ *d*	$\Delta \zeta$	Size	SC$\rho$	SC CIC	Reference
*Allium*	57	4	0.31^a^	128.73^a^	0.34	967	$-5.49$	+0.10	+1.07	27	0.91	282.66	Zanne et al. (2014)
*Asplenium*	133	3	0.76	715.97	0.41	562	$-5.23$	+0.20	+2.00	39	0.86	785.80	Zanne et al. (2014)
Bats	815	29	1.00^a^	7499.95^a^	0.16^c^	30,995^c^	−6.97^c^	+0.01	+0.36	$1.1\times 10^{47}$	0.09	980.05	Shi and Rabosky (2015)
Caryophyllaceae	224	4	0.56^a^	1264.62^a^	0.51	253	$-4.15$	+0.22	+1.67	$4.0\times 10^{14}$	0.77	1470.05	Zanne et al. (2014)
Chameleons	202	6	0.93	1411.94	0.92	14	$-0.83$	0.00	0.00	1	1.00	1440.73	Tolley et al. (2013)
*Eucalyptus*	136	4	NA	NA	0.34	1159	$-5.67$	+0.11	+1.14	27^b^	0.90^b^	840.43	Zanne et al. (2014)
*Euphorbia*	131	6	1.00^a^	847.84^a^	0.32	1467	$-5.50$	+0.04	+0.45	759	0.87	794.71	Zanne et al. (2014)
*Ficus*	112	5	0.90	667.69	0.36	955^c^	−5.25^c^	0.00	+2.56	$7.2\times 10^{7}$	0.29	301.59	Zanne et al. (2014)
*Iris*	137	4	0.54	678.69	0.49	280	$-4.25$	+0.16	+1.19	1	1.00	896.11	Zanne et al. (2014)
Mammals	169	26	0.97	1150.99	0.94	11	0.86	0.00	0.00	1	1.00	1159.65	Meredith et al. (2011)
Primates	372	79	1.00^a^	2993.31^a^	0.37	982	$-2.52$	0.00	0.00	$7.6\times 10^{8}$	0.90	2895.31	Springer et al. (2012)
*Primula*	185	5	0.98	1287.78	0.52	222	$-3.79$	+0.09	+0.56	189	0.93	1258.01	Zanne et al. (2014)
*Ranunculus*	170	6	0.61	948.10	0.35	1082	$-5.20$	+0.04	+0.40	3	0.99	1166.45	Zanne et al. (2014)
*Rhododendron*	117	4	0.85^a^	704.48^a^	0.60	108	$-3.30$	+0.25	+1.70	9	0.97	726.94	Zanne et al. (2014)
Rosaceae	529	7	1.00^a^	4532.15^a^	0.30	2627	$-5.93$	0.00	0.00	$4.6\times 10^{18}$	0.81	4178.25	Zanne et al. (2014)
Scincids	213	6	0.96	1515.92	0.71^c^	61^c^	−2.32^c^	$-0.07$	$-0.49$	1	1.00	1536.24	Rabosky et al. (2014)
*Solanum*	187	6	0.93	1281.97	0.37	939	$-5.05$	+0.06	+0.46	154,125	0.92	1273.22	Zanne et al. (2014)
*Syzygium*	106	3	1.00	651.30	0.58	131	$-3.78$	+0.23	+1.51	1	1.00	651.30	Zanne et al. (2014)
Gymnophiona pre-jackknife	105	24	0.72	546.98	0.47	308	$-2.55$	NA	NA	99	0.85	581.15	This study
Gymnophiona post-jackknife	105	24	0.91	627.87	0.47	307	$-2.55$	NA	NA	1	1.00	643.61	This study
Gymnophiona removed taxon	104	24	0.86	609.09	0.47	308	$-2.55$	NA	NA	3	0.99	634.35	This study
Gordon supertree	14	2	0.77	31.49	0.68	38	$-2.95$	NA	NA	7	0.77	31.49	Gordon (1986)

Note: The calculations reported in this table include the taxon coverage density (*d*), tree resolution ($\rho$), and cladistic information content (CIC) of the terraces’ and ST’s SC, minimum loci required for decisiveness (*k*_min_), and gene sampling sufficiency ($\zeta$).

^a^ Steepest descent selected. SC based only on best scoring trees, not all saved trees.

^b^ Supertree search failed due to memory allocation errors. Terrace based on Dobrin et al.’s (2018) input tree.

^c^ We were unable to replicate Dobrin et al.’s (2018) results despite identical matrix. Data set will be omitted from discussion of results.

### Gene Sampling Sufficiency, Terrace Size, and Supertree Resolution

Gene sampling sufficiency ($\zeta$) was computed for the caecilian data and for a selection of the data sets tested for terrace size by Dobrin et al. (2018) using the latter’s approximation of the expression in part (i) of Steel’s (2016) Proposition 4.22. In order to compute sampling sufficiency, it is first necessary to calculate a theoretical minimum number of loci, *k*_min_, that must be sampled to ensure that taxon coverage, *S*, would be decisive for tree *T*. The *k*_min_ calculation assumes random taxon coverage, and, with the assumptions that all partitions yield binary trees and that trees do not conflict, need not be computed if any locus has been sampled for all taxa, because that is a sufficient condition for *S* to be decisive (Sanderson et al. 2010; Steel 2016). To calculate *k*_min_, we need to first compute the taxon coverage density for the data set (*d*), or the average proportion of taxon sampling, with


(1) \begin{eqnarray*}
{\it d} = \frac{\sum \frac{{\it n}_{l}}{{\it n}}}{{\it k}},
\end{eqnarray*}

where *n*$_{l}$ is the number of taxa sampled in a locus, *n* is the total number of sampled taxa, and *k* the number of sampled loci. Following Dobrin et al. (2018), we calculated *k*_min_ using the approximation:


(2) \begin{eqnarray*}
{\it k}_{min} \approx \frac{\mathrm{ log}\frac{n^{3}}{6p}}{-\mathrm{ log}(1-d^{4})},
\end{eqnarray*}

where the new term *p* is the desired confidence level on the decisiveness of *S* (i.e., the “*P*-value”). The latter was set to 0.05 for all analyses, to allow for direct comparison between the new results and Dobrin et al.’s (2018). With *d* and *k*_min_ computed, we can finally calculate gene sampling sufficiency with


(3) \begin{eqnarray*}
\zeta = -\mathrm{ ln}\frac{{\it k}}{{\it k}_{min}}.
\end{eqnarray*}

A value of $\zeta \ge 0$ means enough loci have been sampled to achieve taxon coverage decisiveness, and $\zeta < 0$ means that more loci must be sampled.

Following from the extension of terraces to the supertree context (Sanderson et al. 2020; Farah et al. 2021), we explored the terrace space associated with the supertrees of the reanalyzed data sets and the caecilian trees with *terraphy* (Zwickl 2014), which allows for the testing of polytomous trees (for analyses exclusively on binary trees see *terraphast*, Biczok et al. 2018). Given a partitioned alignment and a rooted tree, *terraphy* uses Constantinescu and Sankoff’s (1995) supertree algorithm to count (and memory permitting generate) all trees in the terrace induced by the partitions’ taxon coverage; the SC of these trees is then obtained with the Constantinescu and Sankoff (1995) and Steel (1992) algorithms (for further functionalities, see Dobrin et al. 2018). Although terraces have been primarily explored under the concatenated/supermatrix analyses context (Sanderson et al. 2015; Dobrin et al. 2018), it is not insensible to assume that supertree analyses also recover such features of tree space. Particularly, when such supertrees are recovered with MRP and parsimony, analyses have been shown to generate terraces (Sanderson et al. 2015). For the *terraphy* analyses, one of the inferred most parsimonious trees (MPTs) was used as the input tree, allowing for a comparison between the SC of the theoretical terrace space and the SC of the MRPs. The use of an MPT also allows for a comparison between the terrace sizes and resolution found by Dobrin et al. (2018) and those found when partitions with fewer than four sampled taxa are removed from concatenated data sets. Given that the majority of input trees provided/used by Dobrin et al. (2018) were binary, our use of (mostly) fully resolved trees should not artifactually decrease terrace size compared with their analyses, with any change in terrace size due primarily to changes in leaf set size and (in)effective overlap between loci partitions. Lastly, we computed the cladistic information content (CIC, Thorley et al. 1998) for the SC trees of the terrace and MRP analyses.

## Results

### Phylogenetic Analyses

Details of the individual alignments after masking with GBlocks are summarized in Table 2. The taxon coverage density (see Equation 1) for this data set was 0.47, with 16S rRNA having the highest (d = 0.94) and 18S rRNA the lowest (d = 0.09) proportions of taxonomic coverage.

**Figure 3.**
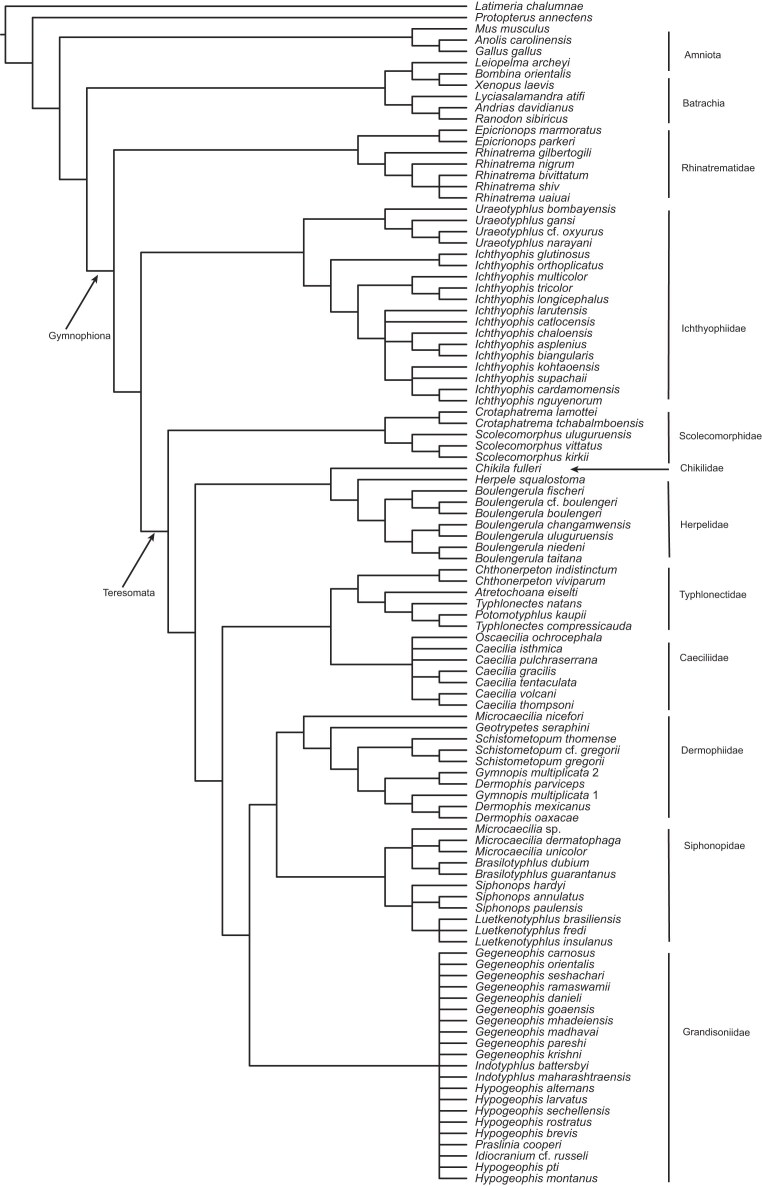
Strict consensus of the MRP supertree inferred from the set of caecilian gene-trees obtained prior to taxonomic instability analyses.

The initial supertree analysis yielded a reasonably well-resolved caecilian phylogeny, except for the relationships within Grandisoniidae, which are completely unresolved in the SC (Fig. 3). Inferred relationships are broadly congruent with recent molecular phylogenetic studies (Pyron and Wiens 2011; Kamei et al. 2012; San Mauro et al. 2012; Pyron 2014; San Mauro et al. 2014; Jetz and Pyron 2018; Acosta-Galvis et al. 2019; Maciel et al. 2019). The lack of resolution within Grandisoniidae and absence of the previously inferred well-supported splits between Indian (*Indotyphlus* + *Gegeneophis*) and Seychelles (*Praslinia* + *Hypogeophis*) grandisoniids (e.g., San Mauro et al. 2004; Kamei et al. 2012) is suggestive of the presence of at least one rogue taxon.

### Gene-Tree Jackknife Analyses Identify Realistic Candidates for Targeted Sequencing

**Figure 4.**
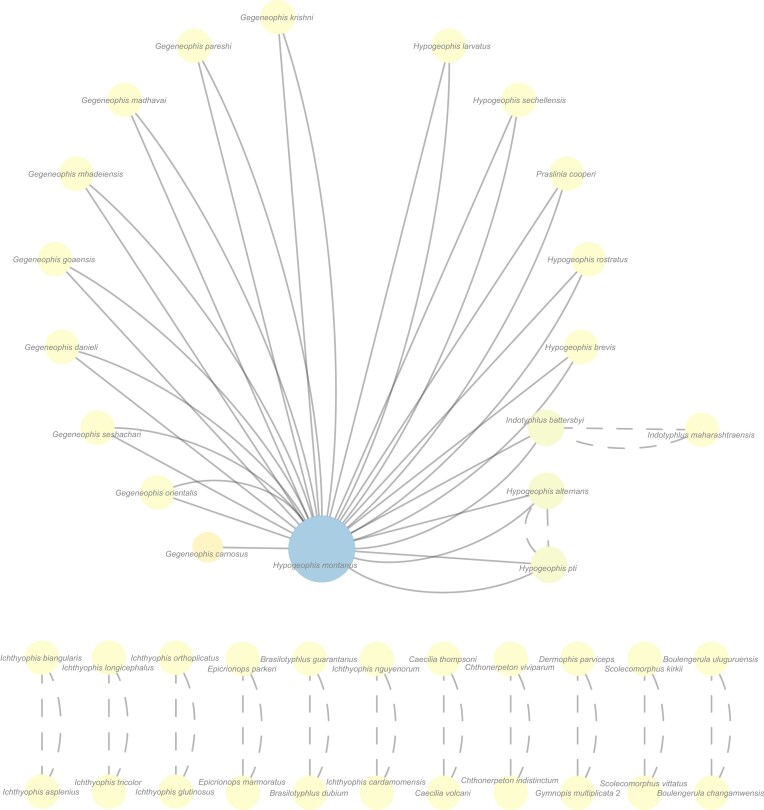
Concatabomination network of the Gymnophiona supertree with 64 stable, unconnected taxa omitted. Full lines correspond to D-type taxonomic equivalences and dashed lines to the symmetrical C/E equivalences. Color and size of nodes are representative of the number of taxonomic equivalences identified for a taxon. The higher the number of D-type taxonomic equivalents is, the more unstable the taxon is inferred to be (following from Siu-Ting et al. 2015).

The concatabominations analysis, including all Gymnophiona gene-trees, found a cluster of instability with 17 taxa anchored by the Seychelles caecilian *Hypogeophis montanus* Maddock, Wilkinson, and Gower, 2018, a further 11 dyads (pairs of linked taxa) and 64 singletons (Fig. 4). The dyads correspond to cherries/sister taxa and are unproblematic, but the cluster connected by *H. montanus* is indicative of ineffective overlap and suggests that *H. montanus* might usefully be deleted, either *a posteriori* by pruning from the individual MRP supertrees or *a priori* from the concatenated alignment.

**Figure 5.**
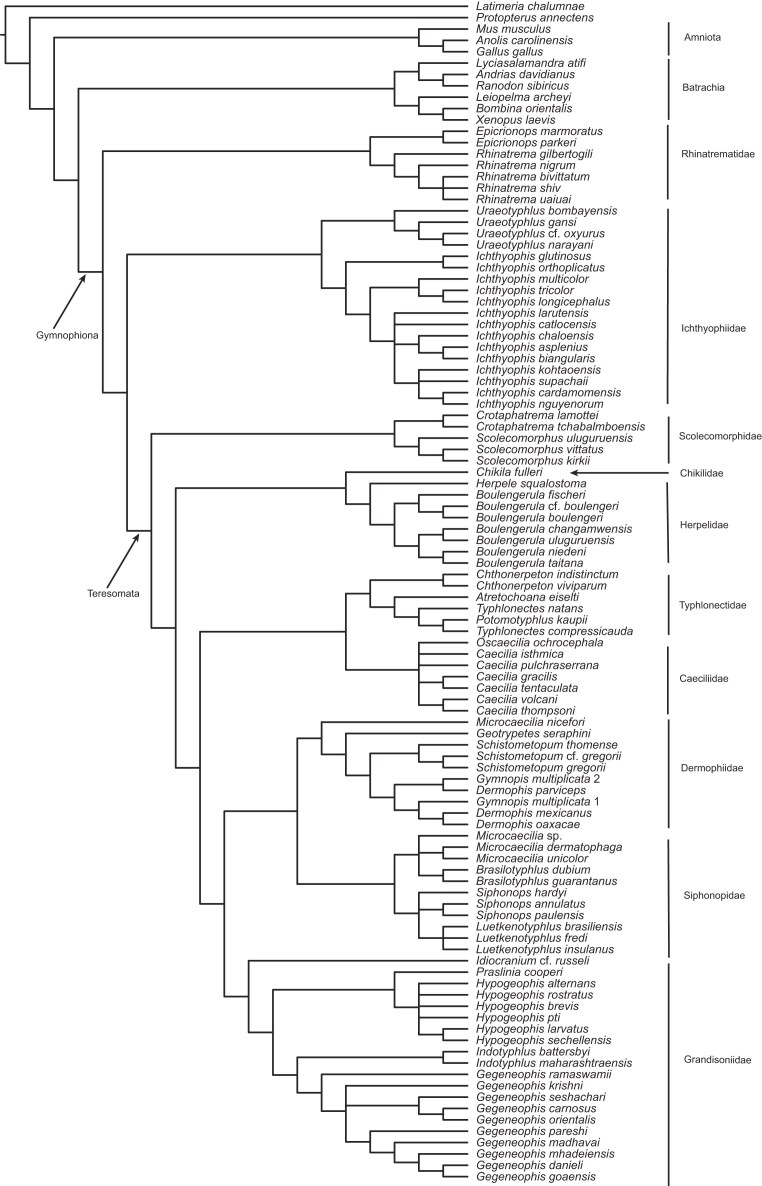
Strict consensus MRP supertree of the caecilian gene-trees inferred with MrBayes omitting *Hypogeophis montanus*.

In fact, both pruning approaches led to the same far better resolved MRP supertree (Fig. 5) and confirm that *H. montanus* is behaving as a rogue taxon. The resulting supertree is reasonably well resolved ($\rho$ = 0.86, $\rho$ is equivalent to the normalised consensus fork of Colless 1980) and recovers the split between Indian and Seychelles grandisoniids. Further confirming *H. montanus* as a rogue taxon, which inspection of the taxonomic complement of the gene-trees reveals to be due to ineffective overlap: *H. montanus* is present in only one of 24 gene-trees (nucDNA locus BDNF; Tables S1–S2) in which the four *Hypogeophis* Peters, 1880, species sampled form a polytomy.

Gene-tree jackknifing identified six gene-trees, which, when removed, led to increased taxonomic instability, marking them as candidates for any targeted increase in taxon sampling (Table 3). These candidate loci are the mitochondrial loci 12S and 16S rRNA, COX1, CYTB and ND1, and the H3A nuclear locus, with removal of the 16S gene-tree showing the largest increase in taxonomic instability (i.e., the best candidate locus). Although it might be expected that all candidate loci correspond to the most taxon-rich loci (true for four of the six candidate loci), RAG1 and ND2 both have higher taxon sampling than ND1 and H3A is one of the least taxon-rich loci (25 of 105 taxa). Thus, taking the existing taxon sampling into account, only four of the candidate loci are realistic targets for increased sampling: 12S and 16S rRNA, COX1 and CYTB, with 16S rRNA being the most promising candidate for increased taxon sampling.

Preliminary sequence alignments revealed a series of *Hypogeophis* spp. DNA sequences (MH055413–MH055428, Maddock et al. 2018) labeled as 12S rRNA that aligned poorly with the other 12S rRNA sequences, which led to the removal of these sequences from the 12S rRNA alignment. However, upon further inspection it was revealed that the sequences were in fact mislabeled 16S rRNA sequences (confirmed with BLAST [Altschul et al. 1990] and by S. T. Maddock, personal communication, 11 December 2019), with MH055425–MH055428 corresponding to *H. montanus* sequences. Thus, through serendipity, sequence data were available for the target taxon/locus pair identified by the gene-tree jackknife analyses.

**Figure 6.**
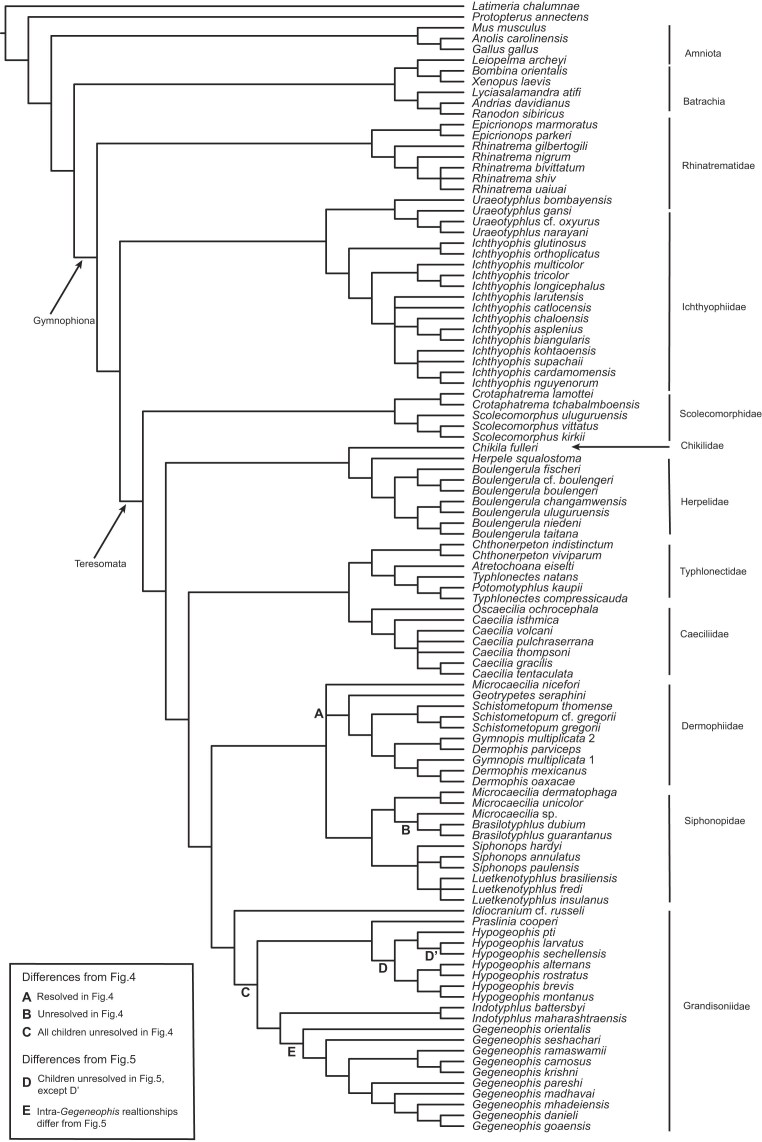
Strict consensus MRP supertree of the caecilian gene-trees inferred with MrBayes after taxonomic instability analyses.

After the addition of data for *H. montanus*, the 16S rRNA realignment had a length of 1142 bp and the concatabominations pipeline yielded a network made up entirely of singletons and dyads (again consisting of cherries), meaning that all, or most, taxa are taxonomically stable. Additionally, tree resolution increased (no *H. montanus* 16S rRNA, $\rho =0.72$; with *H. montanus* 16S rRNA, $\rho =0.91$), showing that the effects of ineffective overlap were successfully mitigated (Fig. 6). The marked increase in tree resolution, including recovery of the split between the Indian and Seychelles clades, after the addition of *H. montanus* data to the 16S rRNA alignment, shows that gene-tree jackknifing is a useful tool for identifying taxa with poor overlap between loci, as well as which loci contribute the most to the inferred (supertree) topology. Interestingly, *H. montanus* is recovered as sister to *Hypogeophis brevis* Boulenger, 1911, which is consistent with Maddock et al.’s (2018) and Sherlock et al.’s (2024) results. Polytomies outside Grandisoniidae were not affected by the inclusion of 16S rRNA data for *H. montanus*, but the changes to the 16S alignment affected relationships within the Dermophidae Taylor, 1969+Siphonopidae Bonaparte, 1850 clade.

Compared with the taxon pruning approach(es), the supertree inferred after the addition of *H. montanus* data is not only more resolved, it also imparts greater information. The tree obtained from pruning *H. montanus* is 53.20 bits more informative than the pre-jackknife supertree, whereas the post-jackknife tree is 62.46 bits more informative than the latter and 9.26 bits more informative than the pruned tree (Table 4).

We also added pseudo-data for *H. montanus* to the other candidate loci (12S rRNA, COX1, CYTB, H3A, and ND1), to all nuclear non-candidate loci (18S rRNA, 28S rRNA, CXCR4, NCX1, RAG1, SIA, and SLC8A3) and to two mitochondrial noncandidate loci (COX2 and ND2), by grafting *H. montanus* onto the tree for concatabominations analyses or by adding a nucleotide string to the supermatrix for the *terraphy* analyses. For each locus of interest, *H. montanus* was always grafted as sister to its nearest sampled relative, following the topology in Figure 6. The concatabominations analyses on the pseudo-data trees showed that all identified candidate loci were reasonable alternatives to 16S rRNA, except for H3A (Table 3), with the number of edges reducing from 59 to 27 and yielding terraces with three trees. Adding pseudo-data to the H3A locus not only did not decrease instability in the concatabomination network, it yielded the original terrace size of 99 trees. The noncandidate 28S rRNA and SIA loci yielded the same concatabomination network and terrace sizes as H3A; their inferred trees were, however, considerably less resolved than H3A’s. Interestingly, “adding” *H. montanus* to 18S rRNA, COX2, CXCR4, NCX1, ND2, RAG1, and SLC8A3, which were not identified as candidate loci, decreased instability in the network. These analyses did, however, yield distinct size terraces; 18S rRNA and SLC8A3 recovered the original 99-tree terrace, CXCR4 and NCX1 yielded a smaller terrace with 81 trees, ND2 and RAG1 yielded a terrace of size nine, and COX2 had a terrace of size three. That most noncandidate loci performed better than H3A can be easily explained by there being more grandisoniids sampled in COX2, NCX1, ND2, and RAG1. The mismatch between reduction in the number of edges in the concatabomination networks and terrace sizes, given the same increase in taxon sampling, suggests that presence/absence matrices (input for *terraphy*) are losing phylogenetic information that is retained by the concatabominations pipeline. If we were to take reduction in terrace size as the measure of how well concatabominations is at identifying taxa/loci for targeted sampling, then it could be argued that concatabominations missed a locus (COX2); however, it did identify five very reasonable alternatives. These results highlight that, before any efforts to collect data are undertaken, any identified candidate loci should be assessed for suitability, as should any taxon-rich loci that are not picked up as stabilizing loci.

### Link Between Taxon Coverage, Gene Sampling Sufficiency, and Terrace Size Remains Elusive

Various statistics from the *terraphy* and supertree anaylses of the caecilian data and for the data sets from Dobrin et al. (2018) are given in Table 4. The removal of loci with fewer than four taxa (minimum required for inference of unrooted trees) from the matrices used in Dobrin et al. (2018) resulted in increased taxon coverage density (*d*) for all data sets except Chameleons, Mammals, and Primates where there was no change, and improvement of the gene sampling sufficiencies ($\zeta$), although, except Mammals, no data set meets the $\zeta \ge 0$ requirement for taxon coverage decisiveness (assuming a 5% confidence level, Table 4). This is in broad agreement with Dobrin et al.’s (2018) results. And like them, our recalculated minimum loci needed for decisiveness (*k*_min_) were in the hundreds to thousands of loci, despite many data sets having terraces with fewer than 10 trees (Chameleons, *Iris* L., Mammals, *Ranunculus* L., *Rhododendron* L., and *Syzygium* P. Browne ex Gaertn.).

#### Gene-tree jackknife *versus* gene sampling sufficiency

Terrace size and minimum number of loci are surprisingly large and sampling sufficiency values low when compared with the supertree resolution for several data sets (Table 4); with *Euphorbia* L. and Rosaceae Juss. having binary supertrees and moderately to highly resolved terrace SC ($\rho$ST $= 1.00$; $\rho$TerraceSC $\ge 0.80$), yet having a *k*_min_  $> 1000$ loci. For the Gymnophiona data sets, *k*_min_ was 308 pre-jackknife and 307 post-jackknife, with a sampling sufficiency of $-2.55$ (for both), despite having one locus with $\approx 94\%$ taxon coverage and considerable increases in supertree and terrace SC resolution with the addition of a single DNA sequence ($\Delta \rho$ST $= 0.19$; $\Delta \rho$Terrace $= 0.15$). This contrasts with the tree jackknife analyses, which identified six “stabilizing” loci, with four strong candidates for increased taxon sampling, and one unstable taxon (*H. montanus*). Although the addition of a single *H. montanus* sequence to the 16S rRNA alignment leads to an increase in supertree resolution and to concatabominations no longer finding unstable taxa, in the sampling sufficiency analyses this only leads to *k*_min_ decreasing by one locus.

For the data sets used in Dobrin et al. (2018), tree jackknife analyses yield results similar to those reported for caecilians, although the effects of targeted increased taxon sampling were not tested. However, unlike the hundreds to thousands of loci identified by gene sampling sufficiency, tree jackknifing identified a maximum of four candidate loci for increased taxon sampling (Table 5). Additionally, no more than three unstable taxa were identified per data set, except for Rosaceae, which had twice as many taxa as the next most speciose data set and the second lowest taxon coverage density of all analyzed data sets, and Bats, which had both the most speciose data set and the lowest taxon coverage density of all data sets. With few exceptions (*Euphorbia, Primula* L., Rosaceae, *Solanum* L., and *Syzygium*), the SCs of the MRP trees of the Dobrin et al. (2018) data sets were less resolved than the SC trees of the theoretical terraces. As in Dobrin et al. (2018), we found terrace sizes ranging over multiple orders of magnitude, from 1 to over $4 \times 10^{18}$ trees. But, compared with their original analyses, terrace sizes either decreased or remained unchanged, with the exception of Caryophyllaceae Juss. and Primates, where terrace size increased by two orders of magnitude. Interestingly, the addition of a single DNA sequence to the caecilian data set (*H. montanus*’s 16S rRNA) led to a one order of magnitude decrease in terrace size—a pattern that was also found for most of the pseudo-data analyses on the candidate caecilian loci. Much like in the caecilian alignments, none of the reanalyzed data sets included a locus with full taxon coverage and the stabilizing loci are not a one-to-one match to the most taxon-rich loci. Thus, tree jackknifing again identifies reasonable numbers of loci and taxa that can be targeted for increased sampling, which are one to three orders of magnitude smaller than the calculated minimum number of loci theoretically required for decisiveness.

**Table 5.** Tree jackknife results for each reanalyzed Dobrin et al. (2018) data set.

Data set	Candidate loci	Unstable taxa
*Allium*	rbcL, matK	*Allium siculum, Allium textile, Allium triquetum*
*Asplenium*	rbcL	*Dicksonia sellowiana*
Bats	12Sto16S, ApolipoProtB, BFIB, BRCA1, COI, CYTB, KVNQ4, OPN1SW, PRKCL, RAG1, RAG2, SPTBN, STAT5A, TASLR2, TSHB, TTN, VWF	*Pteropus vetulus, Pteropus capistratus, Pteralopex atrata, Dobsonia pannietensis, Mormopterus beccarii, Molossus ater, Cheiromeles parvidens, Chalinolobus nigrogriseus, Nyctimene certans, Nyctophilus arnhemensis, Pteropus admiralitatum, Pteropus ornatus, Epomophorus minor, Eumops dabbenei*
Caryophyllaceae	ITS, matK, t-RNAs	*Spergularia marina*
Chameleons	All	None
*Eucalyptus*	None	*Eucalyptus lehmannii, Eucalyptus rodwayi*
*Euphorbia*	ITS, matK, t-RNAs	*Euphorbia silvifolia, Euphorbia polyacantha*
*Ficus*	ITS, matK, rbcL, t-RNAs	*Ficus trigonata, Ficus bullenei, Ficus asperula*
*Iris*	All	None$^{a}$
Mammals	ADRB2, BDNF, BMI1, CREM, PLCB4, PNOC, TYR1	None
Primates	12S, 16S, COI, COIII, IRBPexon, ND4L, VWFintron	*Callithrix saterei*
*Primula*	matK, rbcL, t-RNAs	*Primula veitchiana*
*Ranunculus*	ITS, matK	None$^{a}$
*Rhododendron*	matK, t-RNAs	*Rhododendron micranthum*
Rosaceae	18S, matK, t-RNAs, ITS	*Aphanes microcarpa, Crataegus columbiana, Amelanchier alnifolia, Rubus arizonensis, Rosa orientalis, Rosa corymbifera, Geum macrophyllum, Geum aleppicum, Rubus discolor*
Scincids	All	None
*Solanum*	ITS, matK, rbcL, t-RNAs	*Solanum erianthum*
*Syzygium*	All	None^a^

^^a^^Some taxa in triads or rhombi, but their removal does not affect tree resolution.

## Discussion

Interest in large-scale phylogenetics, with many taxa (e.g., Bininda-Emonds et al. 2007; Pyron and Wiens 2011; Wächter and Melzer 2020) and/or loci (e.g., Siu-Ting et al. 2019; Chan et al. 2020; Streicher et al. 2020), has fueled debates as to how best to infer trees (e.g., Gatesy et al. 2002; de Queiroz and Gatesy 2007; Von Haeseler 2012), evaluate their support (e.g., Bininda-Emonds 2003; Wilkinson et al. 2005; Lemoine et al. 2018; Zaharias et al. 2023), and deal with missing data when working with sparse large matrices (e.g., Roure et al. 2012; Streicher et al. 2016; Molloy and Warnow 2017). Our focus here is on the problem of ineffective overlap of the partitions in multilocus data, how it can be detected, and how best we can make overlap more effective. We treat ineffective overlap as a missing data problem and show how the concatabominations technique, originally developed to identify rogue taxa in character data matrices with a focus on STR (Siu-Ting et al. 2015), can be extended and used to investigate ineffective overlap in the MRs of gene-trees. This use of concatabominations seeks to use the phylogenetic information in the gene-trees to find rogue taxa and to guide experimental design. Our main example, using caecilian multilocus data, demonstrates that concatabominations applied to MRs of gene-trees can identify rogue taxa and that, through gene-tree jackknifing, it is possible to identify genes that appear the most promising for targeted sequencing of the rogue taxa so as to produce more effective overlap.

Identification of unstable taxa using available methods (e.g., Wilkinson 1995; Thorley and Wilkinson 1999; Aberer et al. 2012) is most often a prelude or justification for removing rogues and the problems they may cause, resulting in better resolved and/or supported, but incomplete, phylogenetic hypotheses. Thus, if more complete phylogenies are desired, methods that address the missing data problem through data acquisition rather than data removal are needed.

Goldman (1998) and Massingham and Goldman (2000) proposed an information-based approach to identify the most informative loci in a data set, as well as areas of a phylogeny that might benefit from increased taxon sampling. However, this principled method has only rarely been applied to empirical data sets (e.g., San Mauro et al. 2009, 2012), and its computational demands may make it unsuitable for taxon-rich data.

Gene sampling sufficiency (Sanderson et al. 2010; Steel and Sanderson 2010; Steel 2016) is the other main approach that has been developed to investigate effective overlap and aims to test whether, given a parent tree and a taxon coverage pattern, there are enough sampled loci in a partitioned matrix to ensure taxon coverage decisiveness (i.e., the subtrees of the parent tree induced by the data partitions imply the parent tree). Where the existing taxon coverage is not decisive, this approach estimates the minimum number of loci that needs to be sampled to ensure a high probability of decisiveness (Steel 2016; Dobrin et al. 2018; Parvini et al. 2021). It has also been used to explore the formation of terraces due to ineffective taxonomic overlap between data partitions (Sanderson et al. 2011, 2015).

Gene sampling sufficiency assumes uniform random taxon sampling of infinite and equally informative loci (Sanderson et al. 2010; Dobrin et al. 2018), which is either a biological impossibility (infinite loci) or unlikely to be met in empirical data sets (random taxon sampling). One more, indirect, assumption is that there is one unique binary phylogenetic tree for any set of taxa, and thus penalizes gene-tree/species-tree discordance and hard polytomies. Additionally, because gene sampling sufficiency looks at the amount, not the pattern, of overlap, it can lead to instances where sampling is insufficient, even though a fully resolved, and reasonably well-supported, tree is inferred. This can be seen for the Rosaceae data set, where even though the inferred supertree is binary, gene sampling is insufficient ($\zeta = -5.93$), and 2627 loci need to be sampled for decisiveness (Table 4).

The high number of loci needed for taxon coverage decisiveness (as calculated by gene sampling sufficiency) also runs into the problem of how to increase sampling for hard to collect and rare taxa, even if advances in museomics are making it possible to extract molecular data from previously inaccessible specimens (e.g, Colella et al. 2020; Raxworthy and Smith 2021). The large number of “sufficient” loci takes no account of the financial costs of undirected loci-rich sequencing approaches (Zaharias et al. 2020), which might deter work in nonmodel taxa. The same issues impinge on brute force approaches (sequence everything or sequence a barcode for everything) to eliminating ineffective overlap and achieving decisiveness.

The concatabominations with a gene-tree jackknifing approach introduced and illustrated here can be viewed as a middle ground between the data-only gene sampling sufficiency approach (e.g, Steel and Sanderson 2010) and the experimental design approach of Goldman (1998), by relying only on the set of inferred gene-trees, but having the capacity to identify which of those trees promote decisiveness and which contribute rogues in a multilocus/phylogenomic study. Note that this proposed use of gene-trees does not commit a practitioner to exclusive use of only supertree/species-tree methods. We used a supertree method in our examples because of its utility in illustrating the methodological problem that is the focus of this study and not because of any commitment to that method, position in the supertree *versus* supermatrix debate, or intention to produce the currently best estimate of caecilian phylogeny. Rather, we expect ineffective overlap in gene-trees to also reveal ineffective overlap among data partitions in a concatenated supermatrix intended to be analyzed as such, and be potentially useful in both contexts (results from some other alternative analyses of the caecilian data illustrating this are included in the Supplementary Material).

Our approach is concerned especially with efficient experimental design and increasing effective overlap by adding the least possible data. Although taxon coverage might be expected *a priori* to be a good proxy for how good the addition of data for a given gene would be at reducing ineffective overlap (underpinning of decisiveness, Sanderson et al. 2010; Steel and Sanderson 2010), our analyses show that taxon coverage alone may not tell the whole story. The gene-tree jackknifing analyses identified 12S and 16S rRNA, COX1, CYTB, H3A, and ND1 as the gene-trees where increased taxon sampling would be most effective at reducing ineffective overlap, yet the ND2 and RAG1 trees have greater taxon coverage than H3A and ND1, and the pseudo-data analyses showed that H3A was indeed a poor candidate for targeted sampling. That the addition of a single nucleotide sequence (16S rRNA for *H. montanus*) resulted in dramatically increased topological resolution raises the possibility that, by targeting those taxa missing one or more of the “stabilizing” loci, resolution can be further increased at a fraction of the cost (and effort) required to sequence all taxa for all loci, or for the large *k*_min_ yielded by the gene sampling sufficiency calculations, or even by obtaining complete coverage for a single data partition. Considerations of cost-effectiveness and efficiency would be especially important when dealing with data sets containing hundreds or thousands of loci (Zaharias et al. 2020).

**Figure 7.**
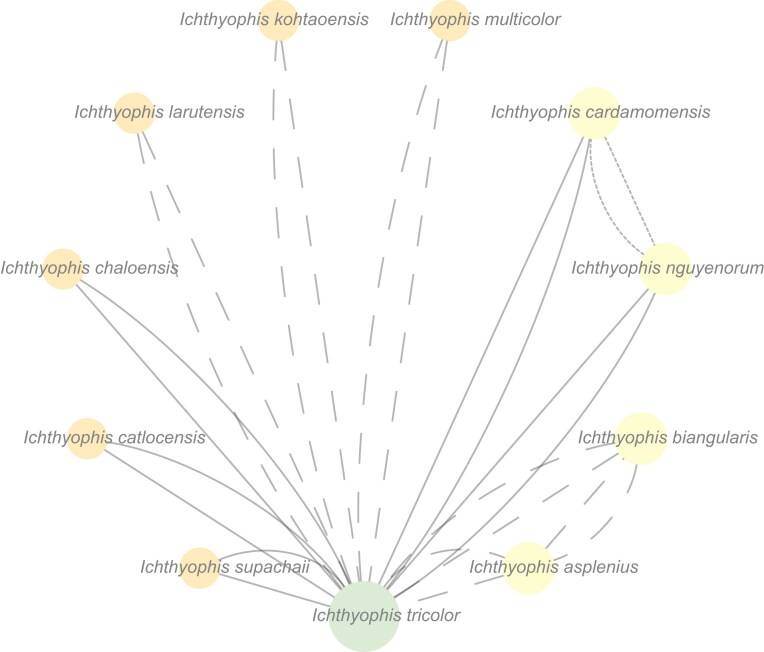
Cluster of within genus instability between *Ichthyophis* species identified after jackknifing of the tree for 16S rRNA. Full lines correspond to D-type taxonomic equivalences, dashed lines to the symmetrical C/E equivalences, and dotted lines to B equivalences. Color and size of nodes are representative of the number of taxonomic equivalences identified for a taxon.

It is noteworthy that additional areas of instability were revealed during tree jackknifing (Fig. 7) of the caecilian multilocus data that may be indicative of what we here call “incomplete” ineffective overlap (i.e., taxa whose overlap is seemingly effective for the full complement of sampled loci, but not for certain subsets of loci). This phenomenon merits further exploration, particularly its cause(s), which might shed light on whether taxa displaying “incomplete” ineffective overlap might be regarded as targets for sequencing of all “stabilizing” loci or windows into what causes a locus to be “stabilizing.” The caecilian data also include instances of taxa with only partial gene sequences (e.g., *Microcaecilia nicefori* (Barbour, 1924)) that may be insufficient to yield well-supported relationships. Of course, because concatabominations and gene-tree jackknifing are heuristics, they are not guaranteed to diagnose ineffective overlap and are not designed to identify issues of low support from partial sequences or data incongruence resulting from evolutionary history. Indeed, resolved trees may be biologically unwarranted or difficult to infer if the evolutionary history includes events such as explosive radiations, incomplete lineage sorting, or hybridization (e.g., Stolzer et al. 2012; Olave et al. 2015). It should also be noted that although ineffective overlap caused by gene presence/absence at the genome level (i.e., genes not shared across taxa) may be identified by the concatabominations pipeline, it cannot be mitigated with any of the approaches discussed in this work. Further, we conjecture that if there is effective overlap, the concatabominations pipeline will not identify spurious rogues and that any edges in the concatabomination network will correspond to “true” equivalents (i.e., to sister taxa).

As currently implemented, the concatabominations pipeline is based on character compatibility and might lose any power to discriminate and thus fail with larger matrices in which the number of pairwise incompatibilities approaches the maximum (Siu-Ting et al. 2015). This issue might be addressed using parsimony in lieu of compatibility, and determining the effect of concatabominations on tree length statistics rather than number of pairwise incompatibilities.

Althoughe we have been able to show that gene-tree jackknifing can be an effective tool for directing targeted sampling, the relationship between terrace size and taxon coverage in empirical data sets remains somewhat elusive (Dobrin et al. 2018; this study). The addition of 16S rRNA data for *H. montanus* had a marked effect on tree resolution and terrace size but no effect on sampling sufficiency (Figs. 3 and 5 and 6 and Table 4). In fact, all three caecilian data sets have $\zeta = -2.55$. It is also worthwhile to note that the caecilian data also lead to similar terrace size patterns when using the concatenated RAxML (Stamatakis 2014) and MrBayes+Astral inferred trees as input to *terraphy* (analytical details and results available in the Supplementary Material). Our reanalyses of the Dobrin et al. (2018) data sets yield a similar lack of pattern between terrace size and sampling sufficiency. Perhaps no pattern can be expected given the implausibility of the assumption of random uniform taxon sampling holding in empirical data sets (Dobrin et al. 2018).

In addition to the pattern that propelled work on terraces, binary inferred trees with terrace size >1 (e.g., *Euphorbia*, Rosaceae), there are also instances where data sets had a terrace size of one but unresolved supertrees (e.g., Chameleons, *Iris*, and post-jackknife caecilians). This is likely a consequence of the *terraphy* analyses not accounting for data incongruence and the resulting topological conflict between partitions (i.e., gene-trees). Lastly, to reduce the computational power and time resulting from *terraphy*’s treatment of polytomies as “soft” and the ensuing enumeration of all possible resolutions (see the “Methods” section in Dobrin et al. 2018), we opted to use MPTs rather than the MRP SCs as input. This decreased terrace sizes but did not affect the terraces’ SCs. An example of this is whether Gordon’s (1986) supertree (Fig. 2) or one of its permitted trees is used as input to *terraphy*. In the former, the analysis yields a terrace with 105 trees, whereas the latter yields the expected seven permitted trees (*terraphast* interestingly yielded nine permitted trees for the same analysis), yet the SC of both terraces is identical. Although it may be argued that choosing a permitted binary tree rather than its supertree artifactually lowers terrace size, it also prevents conflating the effects of ineffective overlap and data incongruence on tree topology.

Because our primary focus is on methods, our analyses of the newly assembled caecilian multilocus data were sufficient to illustrate methods but far from thorough. Nonetheless, it merits some discussion. The inferred supertree (Fig. 6) is in broad agreement with current understanding of caecilian phylogeny and classification (San Mauro et al. 2004) and contains few surprises. Other than the paraphyly of the Siphonopidae and of *Microcaecilia* Taylor, 1968, with respect to the Dermophiidae implied by the phylogenetic placement of *M. nicefori*, all currently recognized families and genera are monophyletic. Alternative phylogenies inferred using supermatrices or with differently inferred gene-trees or different supertree methods (see the Supplementary Material) differ in some details. Different placements of *M. nicefori* including within a monophyletic Siphonopidae or unresolved with respect to Siphonopidae and Dermophiidae underscore the uncertainty that remains in the placement of this species, which is represented only by a partial sequence of a single gene. This is not unexpected given recent findings that reliance on short (non-overlapping) 16S rRNA fragments in amphibian phylogenetics has led to strong, yet inconsistent, support for distinct topologies (Chan et al. 2022). However, given that all partial 16S rRNA fragments used in this study overlap, we have no reason to distrust the gene-trees inferred from the 16S rRNA alignments. Differences in the earliest branching within Teresomata Wilkinson and Nussbaum, 2006, recall the identification of this part of the tree as poorly supported in some previous studies including those employing Goldman’s (1998) approach to experimental design.

Thus, concatabominations with gene-tree jackknifing is a promising approach to tackling taxonomic instability caused by ineffective overlap by identifying those taxa that would benefit from increased locus sampling, as well as the best loci to sample, thus providing a cost-effective, and biologically realistic, alternative to gene sampling sufficiency. Alternatively, this approach might be used to investigate which loci and/or taxa to exclude from computationally intensive analyses without spuriously affecting inferred phylogenetic relationships. However, further work is needed to investigate up to what matrix size (taxa and loci) this approach can be efficiently and/or effectively used.

## Supplementary Material

syaf044_Supplemental_File

## Data Availability

The latest version of the concatabominations pipeline is available from: https://github.com/agalychnica/Concatabominations_v5.

## Supplementary Material

Data available from the Dryad Digital Repository:https://dx.doi.org/10.5061/dryad.9p8cz8wr8.
