# Supplementary material for: Coping with Ineffective Overlap in Multilocus Phylogenetics
Source: Syst Biol. 2025 Jul 3;75(1):52–71. doi: 10.1093/sysbio/syaf044 (PMC12805666; doi:10.1093/sysbio/syaf044)
Supplement: syaf044_Supplemental_File [file syaf044_supplemental_file.pdf]

Supplementary Materials for

Coping with Ineffective Overlap in Multilocus Phylogenetics

Ana Serra Silva[1,2,‡,*], Karen Siu-Ting[3], Christopher J. Creevey[3], Davide Pisani[2,4] and Mark Wilkinson[1]

[1] Herpetology Lab, Science Group, The Natural History Museum, London, SW7 5BD, United Kingdom

[2] School of Earth Sciences, University of Bristol, Bristol, BS8 1RL, United Kingdom

[3] School of Biological Sciences, Queen's University Belfast, Belfast, BT7 1NN, United Kingdom

[4] School of Biological Sciences, University of Bristol, Bristol, BS8 1RL, United Kingdom

[‡] Current address: Department of Genetics, Evolution and Environment, University College London, Gower Street, London WC1E 6BT, United Kingdom

*a.da-silva@nhm.ac.uk, ana.silva.17@ucl.ac.uk

Contents

1 Concatabominations with gene tree jackknife algorithm and example **13**

1.1 Algorithm . . . 13

1.1.1 Note on concatabominations runtime . . . 14

1.2 Example . . . 15

2 Additional Analyses **18**

2.1 Phylogenetic analyses . . . 18

2.1.1 Supertree analyses and candidate loci identification . . 18

2.1.2 Concatenated analyses . . . 19

2.1.3 Effects of new 16S rRNA alignment . . . 19

2.2 Terrace size and resolution . . . 20

3 Results **20**

3.1 Phylogenetics analyses . . . 20

3.2 Terraces . . . 22

4 References **40**

List of Tables

S1 Genbank accessions nuclear loci . . . 5

S1 Genbank accessions nuclear loci (cont.) . . . 6

S1 Genbank accessions nuclear loci (cont.) . . . 7

S1 Genbank accessions nuclear loci (cont.) 8
S2 Genbank accessions mitochondrial loci 9
S2 Genbank accessions mitochondrial loci (cont.) 10
S2 Genbank accessions mitochondrial loci (cont.) 11
S2 Genbank accessions mitochondrial loci (cont.) 12
S3 Runtimes 15
S4 Concatabominations with tree jackknife results RAxML trees . 16
S5 Robin-Foulds distances 21
S6 Concatabominations with tree jackknife results RAxML trees . 23
S7 Terraces 24

List of Figures

S1 Gene tree jackknife example 17
S2 Astral+MrBayes supertree without 16S data for *H. montanus* 25
S3 Astral+MrBayes supertree with 16S data for *H. montanus* . . 26
S4 Astral+MrBayes supertree without data for *H. montanus* . . . 27
S5 Majority-rule consensus of MrBayes tree without 16S data for *H. montanus* 28
S6 Majority-rule consensus of MrBayes tree with 16S data for *H. montanus* 29
S7 Majority-rule consensus of MrBayes tree without data for *H. montanus* 30
S8 Astral+RAxML supertree without 16S data for *H. montanus* . 31
S9 Astral+RAxML supertree with 16S data for *H. montanus* . . 32
S10 Astral+RAxML supertree without data for *H. montanus* . . . 33

S11 RAxML tree of concatenated matrix without 16S data for *H. montanus* 34
S12 RAxML tree of concatenated matrix with 16S data for *H. montanus* 35
S13 RAxML tree of concatenated matrix without data for *H. montanus* 36
S14 Strict consensus of the matrix representation with parsimony tree from RAxML gene trees without 16S data for *H. montanus* 37
S15 Strict consensus of the matrix representation with parsimony tree from RAxML gene trees with 16S data for *H. montanus* . 38
S16 Strict consensus of the matrix representation with parsimony tree from RAxML gene trees without data for *H. montanus* . 39

Table S1: List of taxa used in the caecilian phylogenetic analyses, and (Benson et al., 2008) accessions for nuclear loci. See table S2 for mitogenomes and mitochondrial loci (mt).

**Taxon**	**mt**	**18S**	**28S**	**BDNF**	**CXCR4**	**H3A**	**NCX1**	**RAG1**	**SIA**	**SLC8A3**
Atretochoana eiselti	Y				KX757097	KX757110		KX757122	KX757125	KX757134
Boulengerula boulengeri	Y				EF107484		EF107263	EF107322		EF107425
Boulengerula cf. *boulengeri*	Y									
Boulengerula changamwensis	Y							FR691680		
Boulengerula fischeri	Y							FR691676		
Boulengerula niedeni	Y							FR691679		
Boulengerula taitana	Y						HQ444140	DQ320062		
Boulengerula uluguruensis	Y		DQ283488			DQ284138		FR691681	DQ282670	
Brasilotyphlus dubium	Y									
Brasilotyphlus guarantanus	Y									
Caecilia gracilis	Y				KX757102	KX757108		KX757117	KX757126	KX757142
Caecilia isthmica	Y									
Caecilia pulchraserrana	Y									
Caecilia tentaculata	Y		DQ283717		KX757096	DQ284387		KX757118	KX757130	KX757140
Caecilia thompsoni	Y									
Caecilia volcani	Y						HQ444141	HQ444128		
Chikila fulleri	Y						HQ456773	HQ456772		
Chthonerpeton indistinctum	Y				KX757104	KX757113	EF107266	KX757115	KX757127	KX757138
Chthonerpeton viviparum	Y									
Crotaphatrema tchabalmbaboensis	Y		DQ283676			DQ284342				
Dermophis mexicanus	Y			JQ073153			EF107261	JQ073273		EF107423
Dermophis oaxacae	Y					DQ284428			DQ282897	
Dermophis parviceps	Y									
Epicrionops marmoratus	Y									
Epicrionops parkeri	Y									
Gegeneophis carnosus	Y							HQ444115		
Gegeneophis danieli	Y							HQ444121		
Gegeneophis goaensis	Y							HQ444119		
Gegeneophis krishni	Y							HQ444122		
Gegeneophis madhavai	Y							HQ444123		
Gegeneophis mhadeiensis	Y							HQ444118		
Gegeneophis orientalis	Y							KP400613		
Gegeneophis pareshi	Y							KP400612		
Gegeneophis ramaswamii	Y							AY456255		
Gegeneophis seshachari	Y							HQ444124		
Crotaphatrema lamottei	Y							JN089397		
Geotrypetes seraphini	Y		DQ283662		AY523683	DQ284328	AY523726	DQ320063		EF107361

Continued on the next page

Table S1: *(cont.)* List of taxa used in the caecilian phylogenetic analyses, and GenBank accessions for nuclear loci. See table S2 for mitogenomes and mitochondrial loci (mt).

**Taxon**	**mt**	**18S**	**28S**	**BDNF**	**CXCR4**	**H3A**	**NCX1**	**RAG1**	**SIA**	**SLC8A3**
Grandisonia alternans	Y	GRNRR18S					HQ444143	KP400617		
Grandisonia larvata	Y							KP400618		
Grandisonia sechellensis	Y							KP400619		
Gymnopis multiplicata 1	Y									
Gymnopis multiplicata 2	Y						HQ444144	HQ444132		
Herpele squalostoma	Y		DQ283679		EF107485	DQ284346	EF107264	FR691675		EF107426
Hypogeophis brevis	Y			MH114002				KP400616		
Hypogeophis montanus	Y			MH113997						
Hypogeophis pti	Y			MH114007						
Hypogeophis rostratus	Y	HYORR18S	DQ283524	MT569251	AY948787	DQ284172	AY948829	AY948930	DQ282687	AY948885
Ichthyophis asplenius	Y				EF107480		EF107257	EF107316		EF107419
Ichthyophis biangularis	Y									
Ichthyophis cardamomensis	Y									
Ichthyophis catlocensis	Y									
Ichthyophis chaloensis	Y									
Ichthyophis glutinosus	Y				AY948794		AY948839	AY456256		AY948901
Ichthyophis kohtaoensis	Y	ITYRR18S			EF107451		EF107225	HQ902530		EF107358
Ichthyophis larutensis	Y									
Ichthyophis longicephalus	Y									
Ichthyophis multicolor	Y									
Ichthyophis nguyenorum	Y									
Ichthyophis orthoplicatus	Y				EF107481		EF107258	EF107317		EF107420
Ichthyophis supachaii	Y									
Ichthyophis tricolor	Y									
Idiocranium cf. *russelli*	Y									
Indotyphlus battersbyi	Y							HQ444125		
Indotyphlus maharashtraensis	Y							HQ444126		
Luetkenotyphlus brasiliensis	Y				EF107483		EF107262	EF107321		EF107424
Luetkenotyphlus fredi	Y									
Luetkenotyphlus insulanus	Y									

Continued on the next page

Table S1: *(cont.)* List of taxa used in the caecilian phylogenetic analyses, and GenBank accessions for nuclear loci. See table S2 for mitogenomes and mitochondrial loci (mt).

**Taxon**	**mt**	**18S**	**28S**	**BDNF**	**CXCR4**	**H3A**	**NCX1**	**RAG1**	**SIA**	**SLC8A3**
Microcaecilia dermatophaga	Y									
Microcaecilia nicefori	Y									
Microcaecilia sp.	Y									
Microcaecilia unicolor	Y									
Oscaecilia ochrocephala	Y						HQ444146	HQ444137		
Potomotyphlus kaupii	Y				KX757098	KX757109		KX757121	KX757128	KX757135
Praslinia cooperi	Y				EF107489		EF107269	HQ444138		EF107431
Rhinatrema bivittatum	Y		DQ283700		EF107478	DQ284370	EF107255	AY456257	KX757132	EF107417
Rhinatrema gilbertogili	Y				MH177835	MH177838		MH177842		MH177844
Rhinatrema nigrum	Y						HQ444142	HQ444130		
Rhinatrema shiv	Y									
Rhinatrema uaiuai	Y				MH177834	MH177836		MH177841		MH177840
Schistometopum cf. *gregorii*	Y									
Schistometopum gregorii	Y		DQ283490			DQ284140				
Schistometopum thomense	Y				EF107488		EF107268	HQ444139		EF107430
Scolecomorphus kirkii	Y									
Scolecomorphus uluguruensis	Y				EF107486		EF107265	EF107324		EF107427
Scolecomorphus vittatus	Y		DQ283663		EF107457	DQ284329	EF107232	AY456258	DQ282816	EF107380
Siphonops annulatus	Y						HQ444147	DQ320064		
Siphonops hardyi	Y		DQ283489			DQ284139				
Siphonops paulensis	Y				EF107487	KX757114	EF107267	KX757119	KX757131	EF107429
Typhlonectes compressicauda	Y				KX757099	KX757111		KX757123	KX757129	KX757136
Typhlonectes natans	Y	TYPRR18S	DQ283486	JQ073154	KX757100	DQ284136	EF107229	EF551566		EF107365
Uraeotyphlus bombayensis	Y									
Uraeotyphlus cf. *oxyurus*	Y							AY456259		
Uraeotyphlus gansi	Y									
Uraeotyphlus narayani	Y		DQ283491			DQ284141		DQ282671		

Continued on the next page

Table S1: *(cont.)* List of taxa used in the caecilian phylogenetic analyses, and GenBank accessions for nuclear loci. SIA omitted due to space, none of the species below had sequences available for it. See table S2 for mitogenomes and mitochondrial loci (mt).

**Taxon**	**mt**	**18S**	**28S**	**BDNF**	**CXCR4**	**H3A**	**NCX1**	**RAG1**	**SLC8A3**
**Outgroups**									
Gallus gallus	Y	KT445934	KT445934	NM_001398061	NM_204617		DQ987923	NM_001031188	NM_001293097
Anolis carolinensis	Y	AY859624	AY859623	EU402616	JN702390		GU456076	FJ356739	JF804215
Mus musculus	Y	7CPU_S2	NR_003279		BC031665	BC115816	AF004666	BC138342	AF453257
Xenopus laevis	Y	X04025	X59734	EF035623	Y17895		X90839	XELRAG1X	
Bombina orientalis	Y		DQ283741	EF453367	AY364177	HM998943	AY523715	AY583335	AY948867
Leiopelma archeyi	Y		DQ283588		AY523700	HM998942	HM998951	HM998973	EF107408
Lyciasalamandra atifi	Y			KF645905	KF645581		KF645550	KY342294	KF645521
Ranodon sibiricus	Y	AJ279506		HM037760				HM037735	
Andrias davidianus	Y			EU275889	AY948801		AY948847	AY650142	AY948911
Latimeria chalumnae	Y	LTIRREX	LCU34336			DQ284319			
Protopterus annectens	Y				MH329958			KF911897	

Table S2: List of taxa used in the caecilian phylogenetic analyses, and GenBank accessions for mitogenomes and mitochondrial loci (mtDNA). See table S1 for nuclear loci (nucDNA).

**Taxon**	**nucDNA**	**mitogenome**	**12S**	**16S**	**COX1**	**CYTB**	**ND1**	**ND2**
Atretochoana eiselti	Y		KX757071	KX757082	KX757090			
Boulengerula boulengeri	Y		EF107199					
Boulengerula cf. *boulengeri*	N	GQ244464						
Boulengerula changamwensis	Y		FN652690	FN652722	FR691671	FN652754		
Boulengerula fischeri	Y		FR691654	FR691658	FR691668	FR691663		
Boulengerula niedeni	Y		FN652691	FN652723	FR691670	FN652755		
Boulengerula taitana	Y	AY954504						
Boulengerula uluguruensis	Y	DQ283087	DQ283087	FR691672	FN652748			
Brasilotyphlus dubium	N			MG162601		MG162607		
Brasilotyphlus guarantanus	N			MG162598		MG162606		
Caecilia gracilis	Y	NC_023508						
Caecilia isthmica	N			MN555719	MN555727			
Caecilia pulchraserrana	N			MN555715	MN555723			
Caecilia tentaculata	Y	NC_023507						
Caecilia thompsoni	N			MN555717	MN555725			
Caecilia volcani	Y	GQ244466						
Chikila fulleri	Y	NC_021369						
Chthonerpeton indistinctum	Y	NC_023509						
Chthonerpeton viviparum	N			KU495177	KU494384			
Crotaphatrema lamottei	Y	NC_019596						
Crotaphatrema tchabalmbaboensis	Y		EF219345	EF219346				
Dermophis mexicanus	Y	GQ244467						
Dermophis oaxacae	Y		DQ283455	DQ283455				
Dermophis parviceps	N			EU753994		EU754006		
Epicrionops marmoratus	N	KF540151						
Epicrionops parkeri	N			MN555716	MN555724			
Gegeneophis carnosus	Y		HQ443936	HQ443979	HQ444022	HQ444070		
Gegeneophis danieli	Y		HQ443948	HQ443991	HQ444033	HQ444080		
Gegeneophis goaensis	Y		HQ443943	HQ443986	HQ444029	HQ444075		
Gegeneophis krishni	Y		HQ443954	HQ443997	HQ444039	HQ444085		
Gegeneophis madhavai	Y		HQ443956	HQ443999	HQ444041	HQ444087		
Gegeneophis mhadeiensis	Y		HQ443940	HQ443983	HQ444026	HQ444074		
Gegeneophis orientalis	Y		KP400601	KP400605	KP400611	KP400609		
Gegeneophis pareshi	Y		KP400600	KP400604	KP400610	KP400608		

Continued on the next page

Table S2: *(cont.)* List of taxa used in the caecilian phylogenetic analyses, and GenBank accessions for mitogenomes and mitochondrial loci (mtDNA). See table S1 for nuclear loci (nucDNA).

**Taxon**	**nucDNA**	**mitogenome**	**12S**	**16S**	**COX1**	**CYTB**	**ND1**	**ND2**
Gegeneophis ramaswamii	Y	AY456250						
Gegeneophis seshachari	Y		HQ443970	HQ444013	HQ444055	HQ444101		
Geotrypetes seraphini	Y	AY954505						
Grandisonia alternans	Y	KU974367						
Grandisonia larvata	Y	GQ244470						
Grandisonia sechellensis	Y	NC_023510						
Gymnopis multiplicata 1	N	GQ244471						
Gymnopis multiplicata 2	Y	KF540153						
Herpele squalostoma	Y	NC_019586						
Hypogeophis brevis	Y	KU753817						
Hypogeophis montanus	Y			MH055428				
Hypogeophis pti	Y			MH055418				
Hypogeophis rostratus	Y	GQ244472						
Ichthyophis asplenius	Y	KF540148						
Ichthyophis biangularis	N		AB686145	AB686145		AB686080		
Ichthyophis cardamomensis	N				KP264594	KP264619		
Ichthyophis catlocensis	N				KP264602	KP264626		
Ichthyophis chaloensis	N					KP264625		
Ichthyophis glutinosus	Y	AY456251						
Ichthyophis kohtaoensis	Y	AY458594						
Ichthyophis larutensis	N		AB686156	AB686156		AB686091		
Ichthyophis longicephalus	N			JQ040046				
Ichthyophis multicolor	N		FR715999	FR716007	MG935541	FR716015		
Ichthyophis nguyenorum	N				KP264596	KP264621		
Ichthyophis orthoplicatus	Y		AY101213	AY101233		AY101253		
Ichthyophis supachaii	N				KP264600	KP264624		
Ichthyophis tricolor	N		AF461138	AF461139				
Idiocranium cf. *russelli*	N	KF540156						
Indotyphlus battersbyi	Y		HQ443973	HQ444016	HQ444058	HQ444104		
Indotyphlus maharashtraensis	Y	NC_023512						
Luetkenotyphlus brasiliensis	Y	NC_023513						
Luetkenotyphlus fredi	N		MK660790	MK660785	MK660780			
Luetkenotyphlus insulanus	N			KU495583	KU494790			
Microcaecilia dermatophaga	N	NC_023514						

Continued on the next page

Table S2: *(cont.)* List of taxa used in the caecilian phylogenetic analyses, and GenBank accessions for mitogenomes and mitochondrial loci (mtDNA). See table S1 for nuclear loci (nucDNA).

**Taxon**	**nucDNA**	**mitogenome**	**12S**	**16S**	**COX1**	**CYTB**	**ND1**	**ND2**
Microcaecilia nicefori	N			MN555722	MN555729			
Microcaecilia sp.	N	GQ244473						
Microcaecilia unicolor	N	NC_023515						
Oscaecilia ochrocephala	Y	GQ244474						
Potomotyphlus kaupii	Y	NC_023516						
Praslinia cooperi	Y	NC_023517						
Rhinatrema bivittatum	Y	AY456252						
Rhinatrema gilbertogili	Y		MH177827	MH177830	MH177833			
Rhinatrema nigrum	Y	GQ244468						
Rhinatrema shiv	N		GU566188	GU566189		GU566190		
Rhinatrema uaiuai	Y		MH177826	MH177828	MH177832			
Schistometopum cf. *gregorii*	N	NC_023518						
Schistometopum gregorii	Y		DQ283089	DQ283089				
Schistometopum thomense	Y	GQ244476						
Scolecomorphus kirkii	N			MT433517				
Scolecomorphus uluguruensis	Y		AY450618	AY450625				
Scolecomorphus vittatus	Y	AY456253						
Siphonops annulatus	Y	AY954506						
Siphonops hardyi	Y			DQ283088	KU494789			
Siphonops paulensis	Y		KX757080	EF107203	KX757094	AY954507		
Typhlonectes compressicauda	Y		KX757073	KX757084	KX757092		AY916014	AY916014
Typhlonectes natans	Y	AF154051						
Uraeotyphlus bombayensis	N	NC_023511						
Uraeotyphlus cf. *oxyurus*	Y	AY456254						
Uraeotyphlus gansi	N	NC_023519						
Uraeotyphlus narayani	Y		DQ283090	DQ283090		AY101242		

Continued on the next page

Table S2: *(cont.)* List of taxa used in the caecilian phylogenetic analyses, and GenBank accessions for mitogenomes and mitochondrial loci (mtDNA). See table S1 for nuclear loci (nucDNA).

**Taxon**	**nucDNA**	**mitogenome**	**12S**	**16S**	**COX1**	**CYTB**	**ND1**	**ND2**
**Outgroups**								
Gallus gallus	Y	NC_040970						
Anolis carolinensis	Y	EU747728						
Mus musculus	Y	NC_010339						
Xenopus laevis	Y	MH991335						
Bombina orientalis	Y	NC_006689						
Leiopelma archeyi	Y	NC_014691						
Lyciasalamandra atifi	Y	NC_002756						
Ranodon sibiricus	Y	AJ419960						
Andrias davidianus	Y	KU131042						
Latimeria chalumnae	Y	AB257297						
Protopterus annectens	Y	PRRMTCG						

1 Concatabominations with gene tree jackknife algorithm and example

1.1 Algorithm

This is a simplified algorithm of the concatabominations with gene tree jackknife approach. For details on Step 1 refer to Wilkinson (1995) for taxonomic equivalence and Siu-Ting et al. (2015) for the standard concatabominations pipeline.

For ease of algorithm interpretation, D pairs are pairs of taxa where their pattern of missing data is asymmetric making them potential equivalents. An example would be the taxon pair A (01???11100) and B (01100111??0), with their concatabomination being 0110011100, further examples can be found in the example below.

Step 1:

Given a set of gene trees

Generate its matrix representation (MR)

Identify the taxonomic equivalents (following Wilkinson, 1995) present in the matrix (PerlQ step)

For each D pair *sensu* Siu-Ting et al. (2015) or "potential taxonomic equivalents ... asymmetric both ways" *sensu* Wilkinson (1995)

Combine the two leaves of the D pair into its 'concatabomination'

Add the concatabomination to the data matrix and using character compatibility calculate the pairwise incompatibility of the new matrix

Step 2:

Iteratively remove each gene tree from the set and repeat Step 1 for each gene tree jackknife

Step 3:

Plot the 'concatabomination' networks for the complete tree set and all jackknife replicates

This is done by inputting the file containing all taxonomic equivalences found ('*.taxonomicEqiv.sim') to Cytoscape (Shannon et al., 2003) as a network

For ease of visualisation, remove all edges connecting a taxon to itself ('self-loops' in Cytoscape, NA equivalences in the '*.taxonomicEqiv.sim' file)

To generate the networks shown in the main document follow the protocol provided by Siu-Ting et al. (2015)

Repeat until all networks of interest have been loaded

If the 'concatabomination' network for a jackknife replicate has more edges than the original tree set, it corresponds to a stabilising locus and is a candidate for targeted sampling

1.1.1 Note on concatabominations runtime

Runtimes for the concatabomination pipeline v.5 on the empirical non-jackknifed MRPs ranged from 2s (*Allium*) to 50min (Bats, 39min 22s were spent on the PerlQ step), with all analyses spending the bulk of their runtime on the PerlQ step (table S3). The largest datasets took the longest to run but from the Primates and Rosaceae datasets it is clear that both matrix size and missing data contribute to the runtimes (table S3). All analyses were

Table S3: Runtimes for the Concatabominations pipeline for the Gymnophiona and Dobrin et al. (2018) re-analyses. The times reported correspond to the analyses on the non-jackknifed matrix representations, to obtain total runtimes multiply the time reported by n+1 loci.

**Dataset**	**Taxa**	**MRP characters**	**% Missing**	**Time**	**Reference**
Allium	57	66	50.35	2s	Zanne et al. (2014)
Asplenium	133	154	14.56	5s	Zanne et al. (2014)
Bats	815	3415	64.00	50min	Shi and Rabosky (2015)
Caryophyllaceae	224	443	46.31	3.5min	Zanne et al. (2014)
Chameleons	202	1095	8.08	4.25min	Tolley et al. (2013)
Eucalyptus	136	174	21.51	22s	Zanne et al. (2014)
Euphorbia	131	235	33.69	16s	Zanne et al. (2014)
Ficus	112	184	43.76	9s	Zanne et al. (2014)
Iris	137	254	20.63	19s	Zanne et al. (2014)
Mammals	169	4050	5.89	1.7min	Meredith et al. (2011)
Primates	372	10692	57.69	37min	Springer et al. (2012)
Primula	185	466	27.71	1.4min	Zanne et al. (2014)
Ranunculus	170	343	21.95	39s	Zanne et al. (2014)
Rhododendron	117	271	25.34	18s	Zanne et al. (2014)
Rosaceae	529	1072	41.38	29min	Zanne et al. (2014)
Scincids	213	883	19.04	2.4min	Rabosky et al. (2014)
Solanun	187	392	28.24	57s	Zanne et al. (2014)
Syzygium	106	174	15.81	8s	Zanne et al. (2014)
Gymnophiona	105	929	41.68	28s	This study

run in a virtual machine instance of Ubuntu.18.04.6 LTS, restricted to 5G RAM and one CPU core.

1.2 Example

We started by randomly generating a binary 10-taxon tree (labelled sp1 to sp10), which we used as the parent tree to subsample three partially overlapping compatible 'gene trees' (T1-T3), see figure S1. All 'gene trees'

included the tips labelled sp3, sp5 and sp6. To generate T1 and T3 we randomly sampled four and three tips, respectively, to remove from the parent tree. For T2, we removed those tips kept in T1 to make a pair of trees with ineffective overlap.

Running the MRs for the set with all three 'gene trees' and each of the gene tree jackknives through the concatabominations pipeline shows that removal of either T1 and T2 considerably decreases the amount of taxonomic instability in the dataset (table S4). It also shows that T3, which has three tips in common with T1 and five with T2, would be the candidate 'locus' for targeted sequencing in this dataset as it is the only 'locus' not increasing instability in the dataset. However, T3 cannot be called a 'stabilising locus' as its removal does not increase taxonomic instability in relation to the original dataset, it leads instead to recovering the same instability network as the 'All trees' matrix.

Table S4: Concatabominations with tree jackknife results, number of edges excludes self-loops.

**MR**	**Taxa in network**	**Edges in network**	**Taxa in jackknifed "locus"**
All trees	10	31	NA
no T1	7	5	6
no T2	9	23	7
no T3	10	31	6

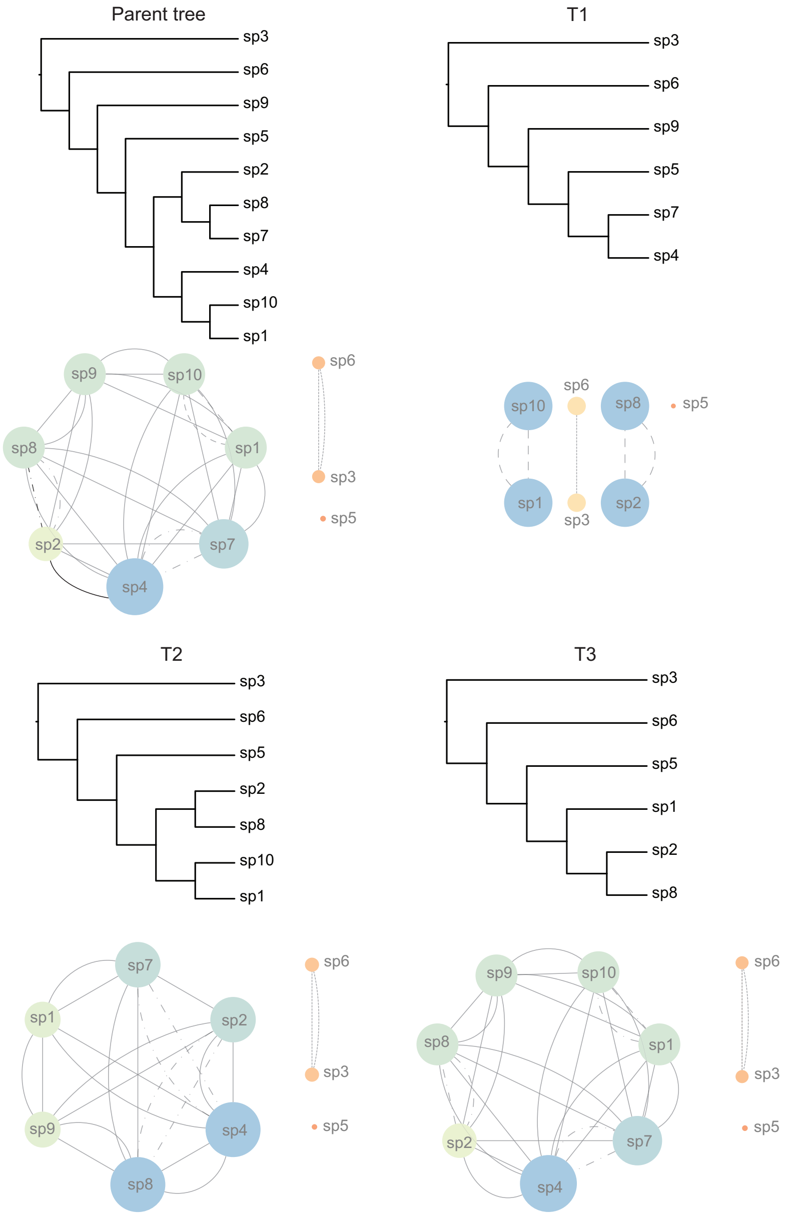

Figure S1: The concatabomination network below each gene tree (T1-T3) corresponds to the analysis of the gene tree jackknife omitting that input tree. The network below the parent tree is that obtained by running the complete example dataset through the concatabominations pipeline. Full lines correspond to D-type taxonomic equivalences, dashed lines to the symmetrical C/E equivalences, dot-dash lines correspond to B equivalences and dotted lines to A equivalences. Colour and size of nodes is representative of the number of taxonomic equivalences identified for a taxon.

2 Additional Analyses

2.1 Phylogenetic analyses

2.1.1 Supertree analyses and candidate loci identification

Following from the molecular data processing detailed in the main text, we used the majority-rule consensuses of the caecilian gene trees inferred with MrBayes v.3.2.7 (Ronquist et al., 2012) as input to the quartet-based summary method Astral-III v.5.6.3 (Zhang et al., 2018), under default settings.

Gene trees were also inferred with RAxML's v.8.2.12 (Stamatakis, 2014) rapid hill climbing algorithm (-f d), with 1000 replicates, under the GTR+I model (to allow comparison between the concatenated analyses described below). No bootstrap analyses were run and the best maximum likelihood trees were used as input to Astral and to standard matrix representation with parsimony (MRP, Baum and Ragan, 2004) analyses. The MRP searches were run in PAUP* v.4.a169 (Swofford, 2003) with 1000 replicates of random addition and 'MulTrees' selected, *Latimeria* Smith, 1939 as the outgroup, and all other parameters under their defaults. As in the analyses detailed in the main text, inferred trees were summarised with the strict consensus (SC, **?**).

These analyses were run with the original 16S rRNA alignment lacking *Hypogeophis montanus*, the 16S rRNA alignment including *H. montanus* and the BDNF alignment with *H. montanus* removed. In other words, *H. montanus* present only in BDNF, *H. montanus* in 16S rRNA and BDNF and *H.*

montanus absent from all alignments.

Concatabominations with gene tree jackknifing analyses were run for the RAxML inferred gene trees following the protocol detailed in the main text.

2.1.2 Concatenated analyses

Partitioned-by-gene concatenated analyses were run using MrBayes and RAxML, with the former running under identical settings to the gene tree searches. Because the trees were being used for comparison purposes only, convergence was checked primarily with the ASDSF measure, with the PSRF measure being allowed a small number (< 5) of parameters that exceeded the recommended value.

For the RAxML analyses, non-parametric bootstrap analyses were run independently of the rapid hill climbing tree search, under the same model (GTR+I) and number of replicates (1000). The bootstrap proportions were projected onto the best maximum likelihood trees with the '-f b' option.

These analyses were run with both 16S rRNA alignments and with a matrix where *H. montanus* was removed from the BDNF alignment.

2.1.3 Effects of new 16S rRNA alignment

For each inference approach, the topological similarity of the trees inferred under the three *H. montanus* sampling scenarios was measured with the Robinson-Foulds tree-to-tree distance (RF, Robinson and Foulds, 1981).

2.2 Terrace size and resolution

As with the analyses detailed in the main text, we used *terraphy* (Zwickl, 2014) to explore the terrace space associated with each inferred caecilian tree. Below we report the computed terrace sizes, terrace strict consensus resolutions (ρ, equivalent to Colless's (1980) normalised consensus fork) and cladistic information content (Thorley et al., 1998) for all phylogenetic analyses not included in the main text. Because the data matrices remain unchanged, all measures related to gene sampling sufficiency (ζ, see main text and Dobrin et al., 2018) are identical to those reported in the main text.

3 Results

3.1 Phylogenetics analyses

The concatenated, Astral and RAxML+MRP analyses (figs. S2-S16) while recovering slightly different topologies tend to differ primarily on teresomatan relationships, intra-generic relationships of *Gegeneophis* and *Ichthyophis*, and on the relationships between Seychelles caecilians. Some interesting differences recovered between methods include the lack of intra-amniote resolution and the placement of *Microcaecilia nicefori* in Grandisoniidae in the concatenated RAxML analysis, likely due to *M. nicefori*'s partial gene sequences.

Based on RF distances, the results of the analyses with the original 16S rRNA and of those completely omitting *H. montanus* were more similar to each other than to those inferred from the updated 16S alignment (table S5).

Table S5: Robinson-Foulds distances for each three-tree set, including the MrBayes+MRP trees described in the main text. T1 corresponds to the original alignments, T2 to the 16S rRNA alignment with *Hypogeophis montanus*, and T3 to the BDNF alignment without *H. montanus*. For the distance between T3 and T1/T2 *H. montanus* was pruned from the latter tree(s).

**MrBayes+MRP**				**MrBayes+Astral**				**MrBayes**			
	T1	T2	T3		T1	T2	T3		T1	T2	T3
T1	-			T1	-			T1	-		
T2	24	-		T2	32	-		T2	17	-	
T3	14	15	-	T3	8	24	-	T3	0	15	-
**RAxML+MRP**				**RAxML+Astral**				**RAxML**			
	T1	T2	T3		T1	T2	T3		T1	T2	T3
T1	-			T1	-			T1	-		
T2	14	-		T2	20	-		T2	4	-	
T3	0	13	-	T3	0	20	-	T3	1	4	-

The concatabominations analyses on the RAxML inferred gene tree did not find *H. montanus* to be unstable, which is easily explained by all inferred trees, including BDNF, being binary and most taxonomic equivalents were C/E pairs. However, gene tree jackknives on the RAxML inferred gene trees still identify 12S rRNA, 16S rRNA and COX1 as "stabilising" loci (table S6) and, thus, the best targets for increased sampling. This shows that even for seemingly stable datasets (the concatenated tree had a terrace size of 31 and the summary tree yielded a 15-tree terrace, table S7) the concatabominations pipeline can identify the best loci to add taxa to if sampling all loci for a target taxon is unfeasible.

3.2 Terraces

The exploration of the theoretical terrace space associated with the additional phylogenetic analyses described above yield patterns that are broadly congruent with those described in the main text (tables S7 and 4). The addition of 16S sequence data for *H. montanus*, or its complete removal from the sequence alignments, results in datasets with lowered levels of ineffective overlap and thus smaller terraces with more resolved strict consensuses. Surprisingly, the terraces associated with the concatenated MrBayes analyses all consist of a single tree when the tree with highest posterior probability is used as input. This would be consistent with Sanderson et al.'s (2015) finding that Bayesian analyses do not generate terraces (although they do yield a posterior distribution of trees, as seen by their partially unresolved consensuses, see figs. S6-8 and table S7). The effects of the targeted taxon sampling are less clear on the inferred trees, but these include polytomous trees, and despite reducing ineffective overlap by adding/removing *H. montanus* sequences, other sources of topological instability, like data incongruence between loci, remain in the data.

Table S6: Concatabominations with tree jackknife results, number of edges excludes self-loops. Candidate loci for targeted taxon sampling highlighted in bold text.

MR	Taxa in network	Edges in network	Taxa in jackknifed locus
All loci	105	19	NA
All loci, no *Hypogeophis montanus*	104	17	NA
**no 12S rRNA**	105	23	84
**no 16S rRNA**	103	21	99
no 18S rRNA	105	19	10
no 28S rRNA	105	19	19
no ATP6	105	19	53
no ATP8	105	19	53
no BDNF	104	17	13
**no COX1**	105	31	86
no COX2	105	19	53
no COX3	105	19	53
no CXCR4	105	19	33
no CYTB	104	19	81
no H3A	105	19	25
no NCX1	105	19	34
no ND1	105	19	54
no ND2	105	19	55
no ND3	105	19	53
no ND4	105	19	53
no ND4L	105	19	53
no ND5	105	19	53
no ND6	105	19	53
no RAG1	105	19	65
no SIA	105	19	13
no SLC8A3	105	19	32
with 16S rRNA for *H. montanus*	105	19	NA

Table S7: Terrace size, resolution (ρ) and cladistic information content for the additional phylogenetic analyses.

	Inferred		Terraces		
**Analysis**	ρ	**CIC**	**Size**	**SC** ρ	**SC CIC**
MrBayes+Astral, 16S no *H. montanus*	0.95	635.69	29	0.86	591.15
MrBayes+Astral, 16S with *H. montanus*	0.96	637.27	1	1.00	643.61
MrBayes+Astral, no *H. montanus*	0.96	629.59	1	1.00	635.93
MrBayes, 16S no *H. montanus*	0.94	635.69	1	1.00	643.61
MrBayes, 16S with *H. montanus*	0.97	640.44	1	1.00	643.61
MrBayes, no *H. montanus*	0.94	628.01	1	1.00	635.93
RAxML+Astral, 16S no *H. montanus*	0.88	621.53	1	1.00	643.61
RAxML+Astral, 16S with *H. montanus*	0.88	623.12	1	1.00	643.61
RAxML+Astral, no *H. montanus*	0.87	613.85	1	1.00	635.93
RAxML, 16S no *H. montanus*	1.00[a,b]	643.61	31	0.86	586.20
RAxML, 16S with *H. montanus*	1.00[a]	643.61	1	1.00	643.61
RAxML, no *H. montanus*	1.00[a]	635.61	1	1.00	635.93
RAxML+MRP, 16S no *H. montanus*	0.80	598.96	15	0.97	638.12
RAxML+MRP, 16S with *H. montanus*	0.83	608.84	1	1.00	643.61
RAxML+MRP, no *H. montanus*	0.80	594.45	1	1.00	635.93

a Based on binary best tree.

b H. montanus *has zero-length branch.*

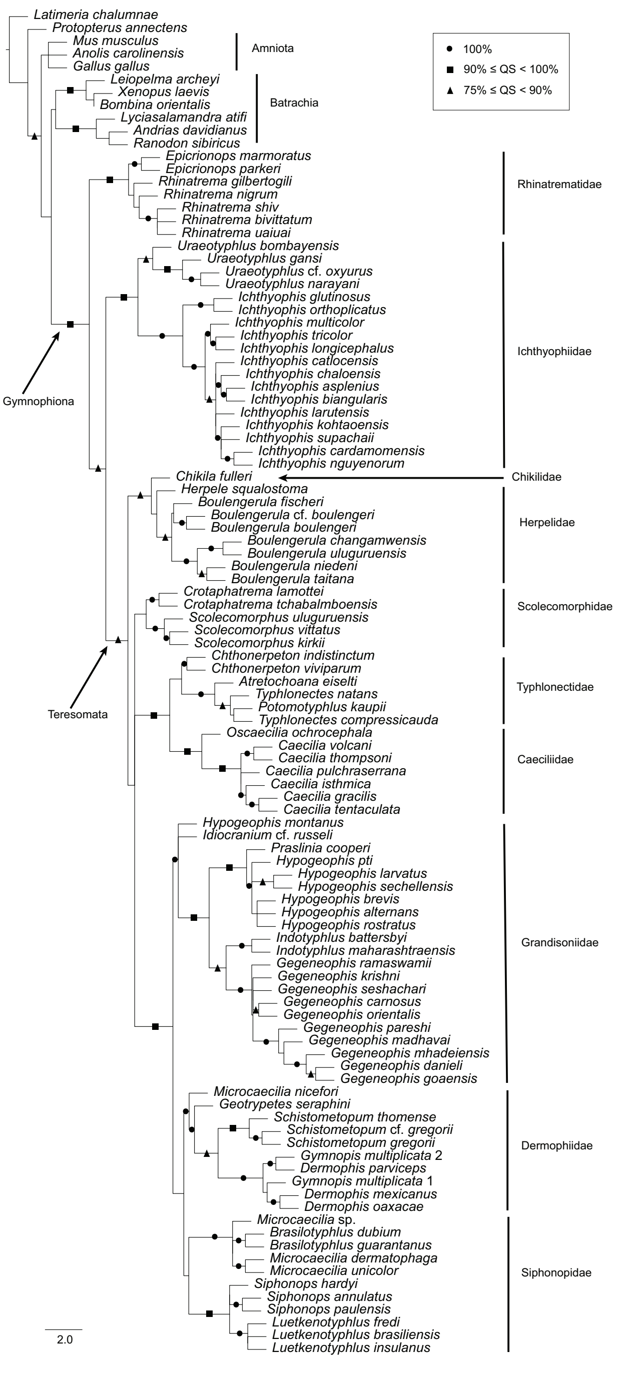

Figure S2: Astral-III v.5.6.3 (Zhang et al., 2018) supertree of the caecilian gene trees inferred with MrBayes v.3.2.7 (Ronquist et al., 2012) without 16S data for *Hypogeophis montanus*. All branches with quartet support (QS) below 50% collapsed. Circles denote branches with 100% QS, squares denote 90% $\geq$ QS $<$ 100% and triangles 75% $\geq$ QS $<$ 90%. Scale bar represents coalescent units.

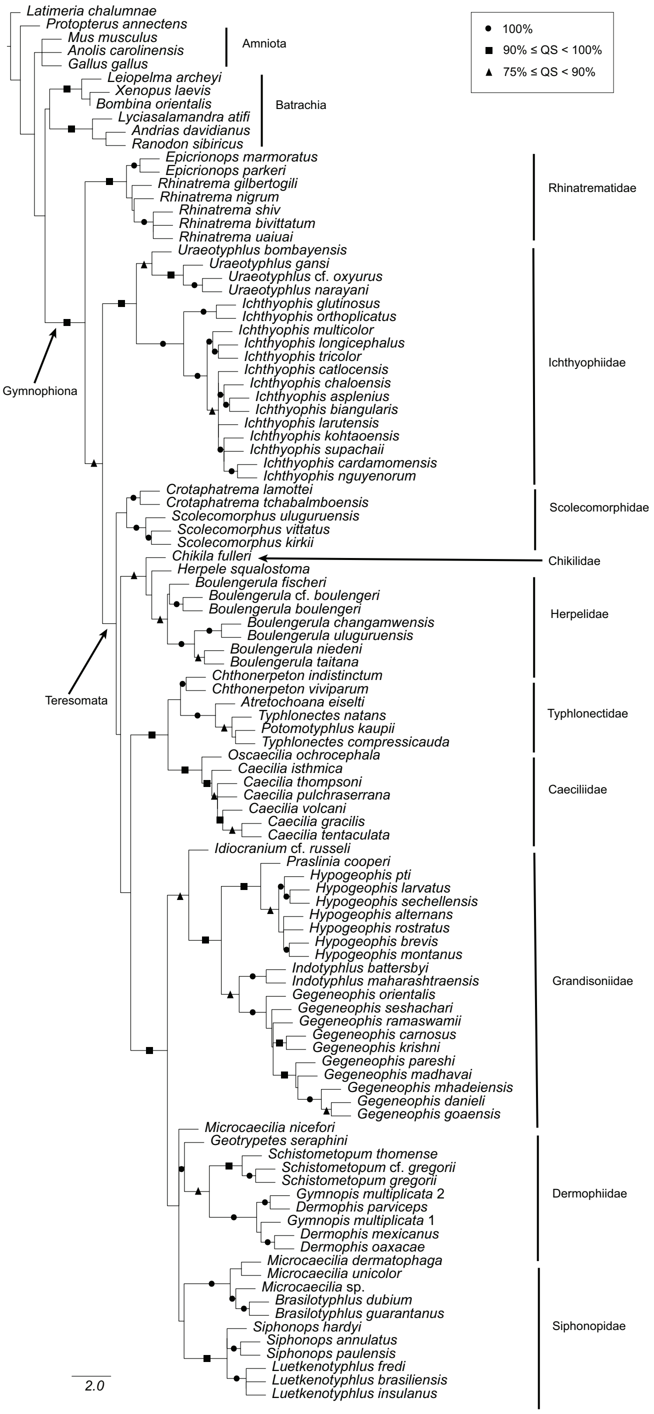

Figure S3: Astral supertree of the caecilian gene trees inferred with MrBayes with 16S data for *H. montanus*. All branches with QS below 50% collapsed. Circles denote branches with 100% QS, squares denote 90% ≥ QS < 100% and triangles 75% ≥ QS < 90%. Scale bar represents coalescent units.

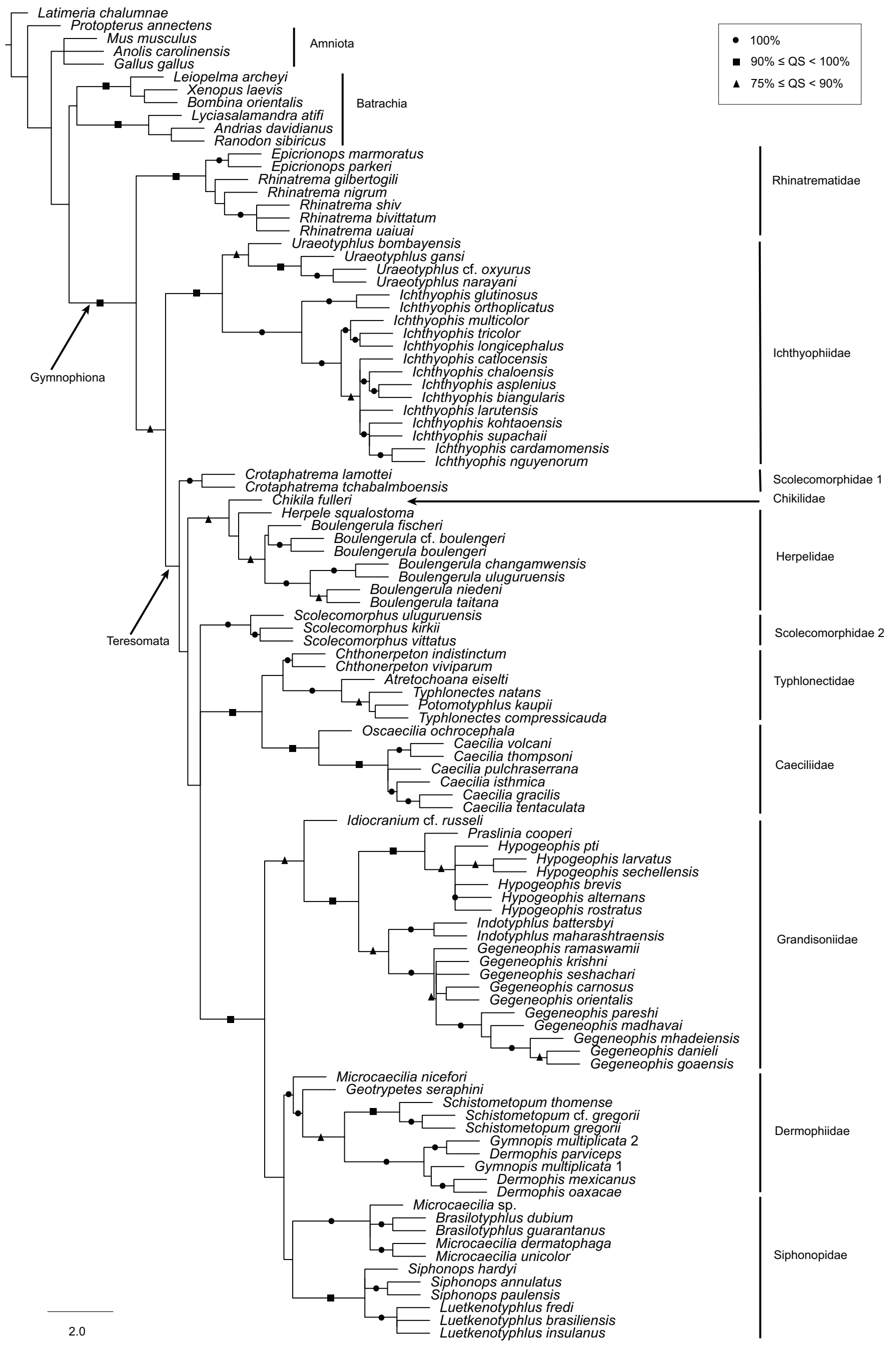

Figure S4: Astral supertree of the caecilian gene trees inferred with MrBayes without *H. montanus*. All branches with QS below 50% collapsed. Circles denote branches with 100% QS, squares denote 90% $\geq$ QS $<$ 100% and triangles 75% $\geq$ QS $<$ 90%. Scale bar represents coalescent units.

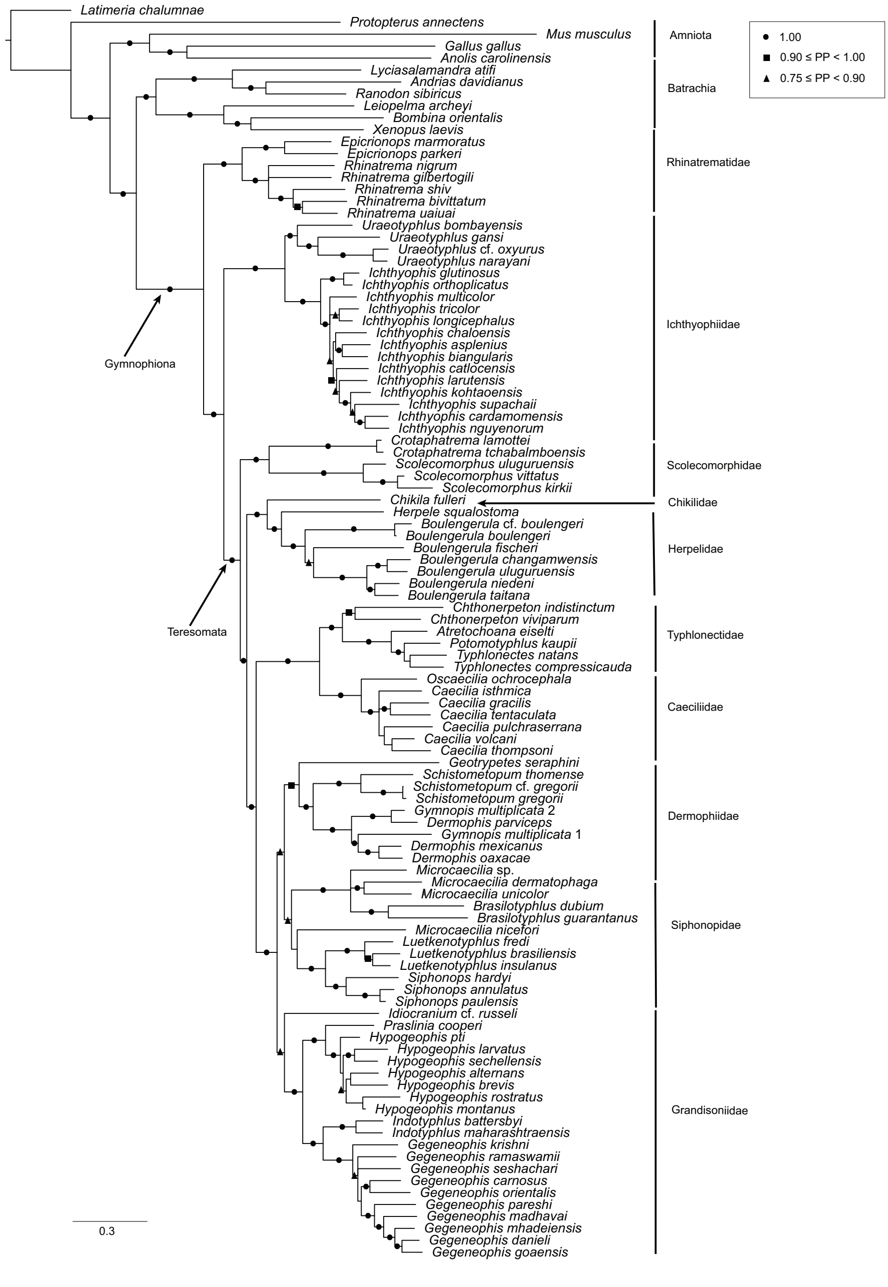

Figure S5: Majority-rule consensus (MRC, Margush and McMorris, 1981) of the MrBayes tree inferred from the concatenated caecilian matrix missing *H. montanus* data for the 16S rRNA locus. All branches with posterior probability (PP) below 0.50 collapsed. Circles denote branches with 1.00 PP, squares denote $0.90 \geq$ PP < 1.00 and triangles $0.75 \geq$ PP < 0.90. Scale bar represents estimated substitutions per site.

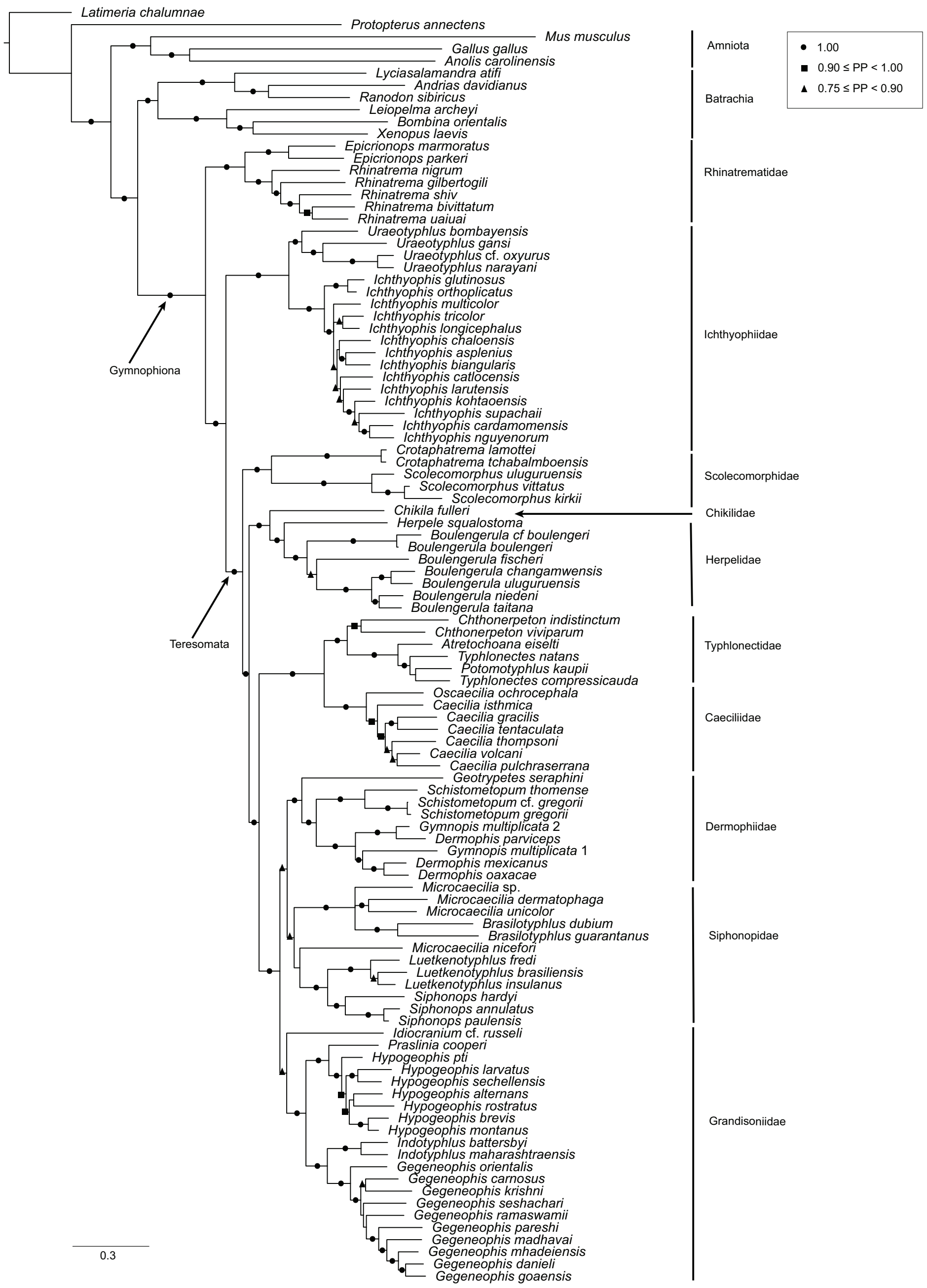

Figure S6: MRC of the MrBayes tree inferred from the concatenated caecilian matrix with *H. montanus* data for the 16S rRNA locus. All branches with PP below 0.50 collapsed. Circles denote branches with 1.00 PP, squares denote $0.90 \geq$ PP < 1.00 and triangles $0.75 \geq$ PP < 0.90. Scale bar represents estimated substitutions per site.

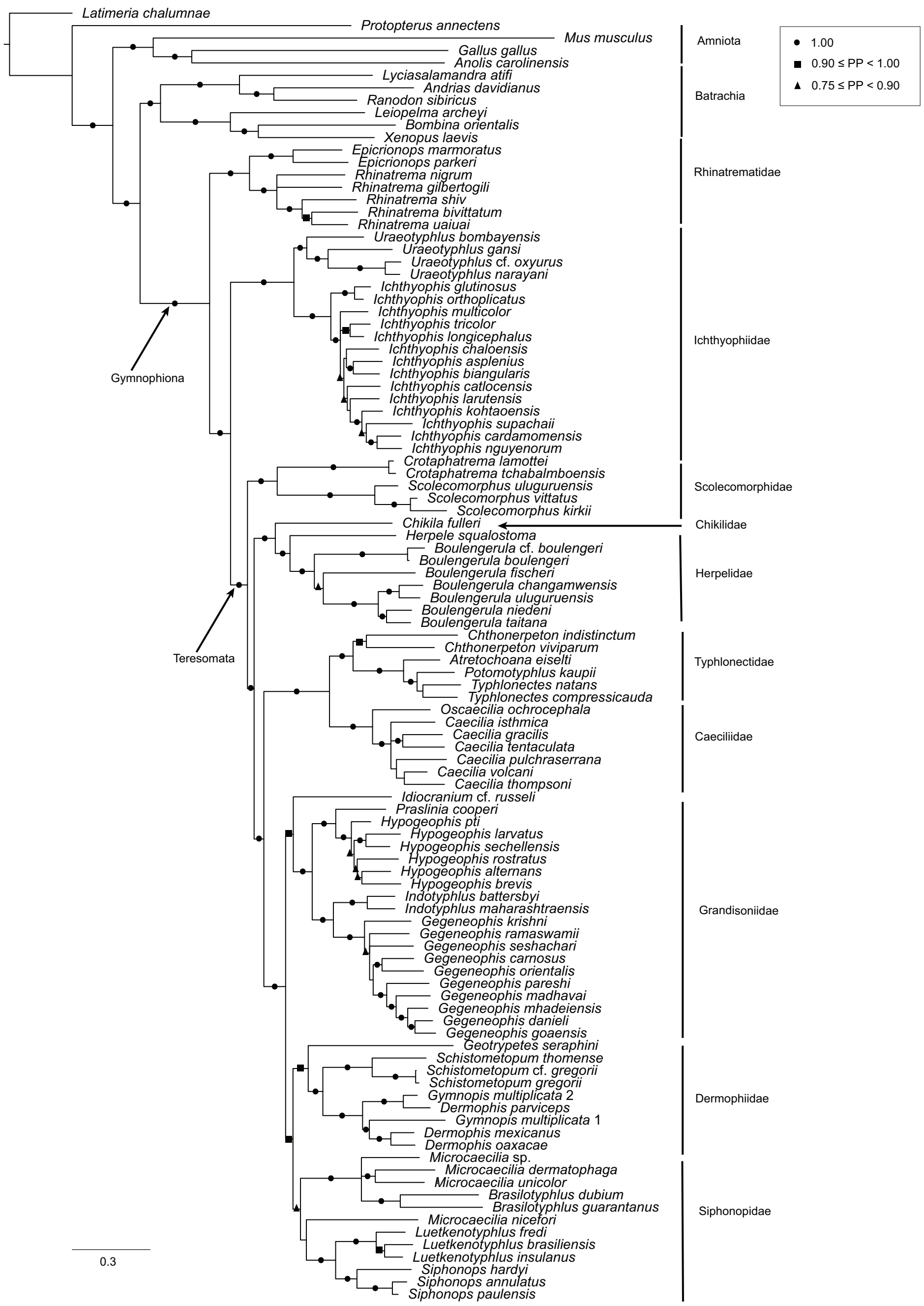

Figure S7: MRC of the MrBayes tree inferred from the concatenated caecilian matrix with no *H. montanus* data. All branches with PP below 0.50 collapsed. Circles denote branches with 1.00 PP, squares denote $0.90 \geq$ PP < 1.00 and triangles $0.75 \geq$ PP < 0.90. Scale bar represents estimated substitutions per site.

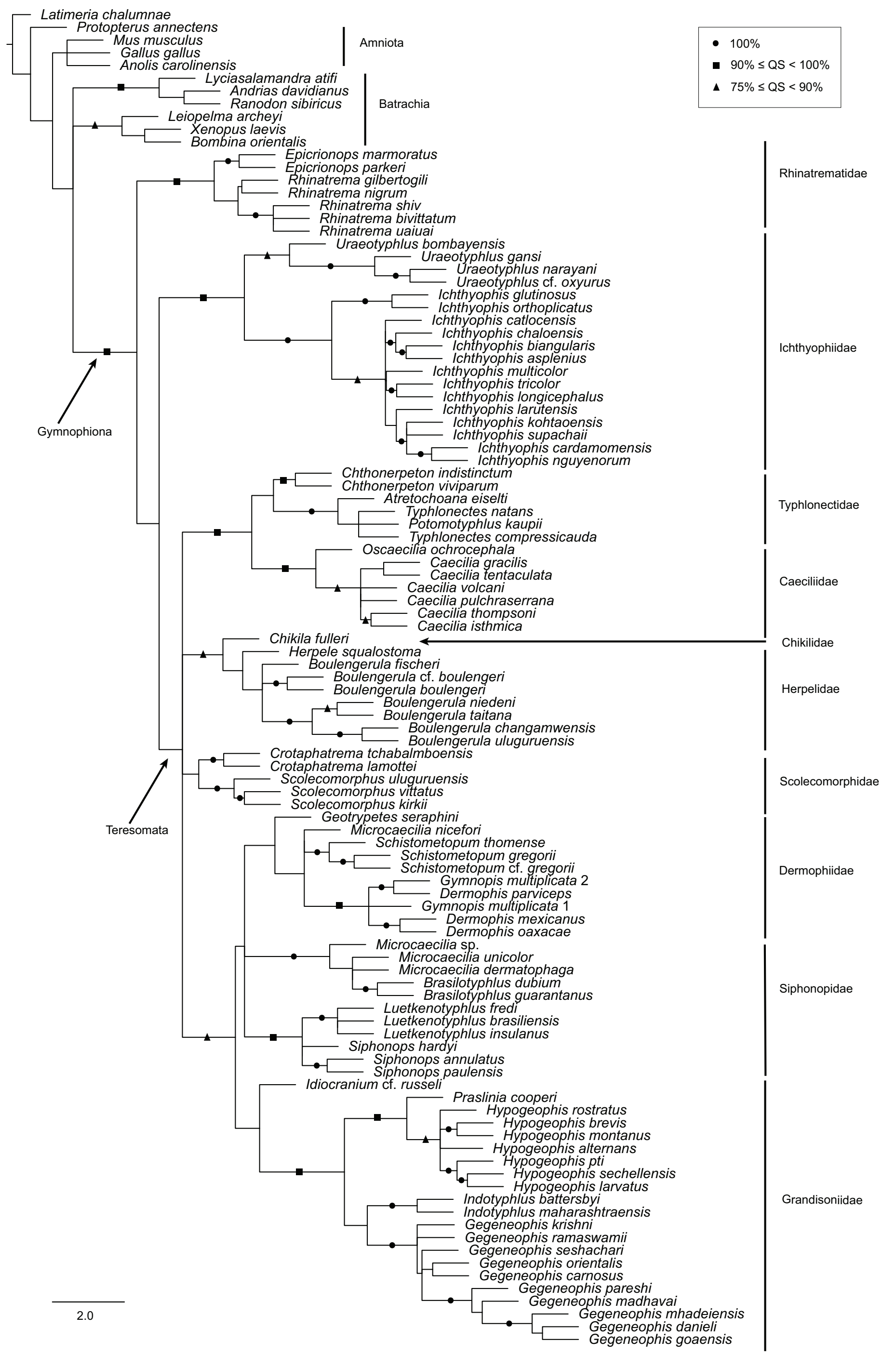

Figure S8: Astral supertree of the caecilian gene trees inferred with RAxML v.8.2.12 (Stamatakis, 2014) without 16S data for *H. montanus*. All branches with QS below 50% collapsed. Circles denote branches with 100% QS, squares denote 90% ≥ QS < 100% and triangles 75% ≥ QS < 90%. Scale bar represents coalescent units.

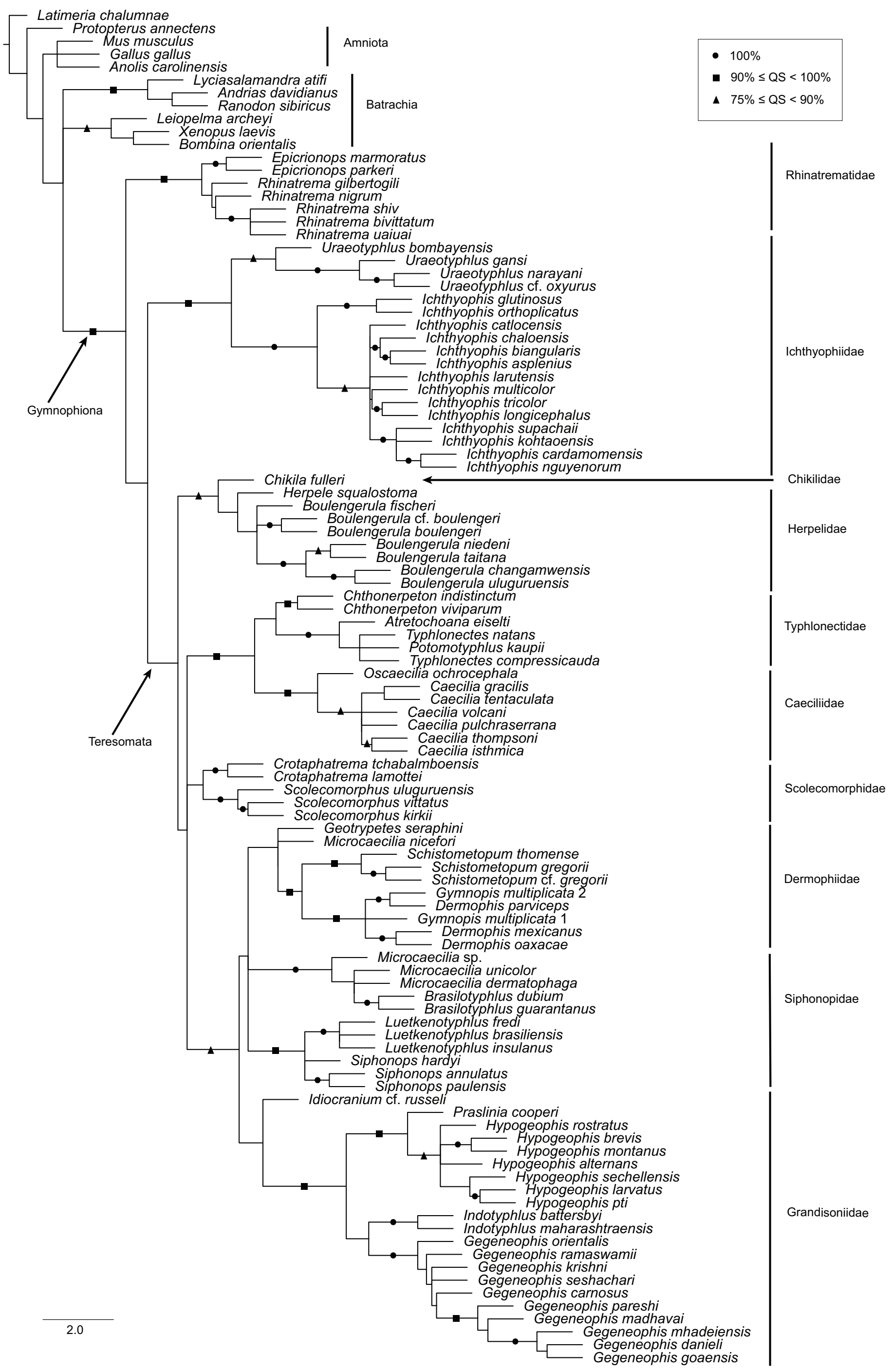

Figure S9: Astral supertree of the caecilian gene trees inferred with RAxML with 16S data for *H. montanus*. All branches with QS below 50% collapsed. Circles denote branches with 100% QS, squares denote 90% $\geq$ QS $<$ 100% and triangles 75% $\geq$ QS $<$ 90%. Scale bar represents coalescent units.

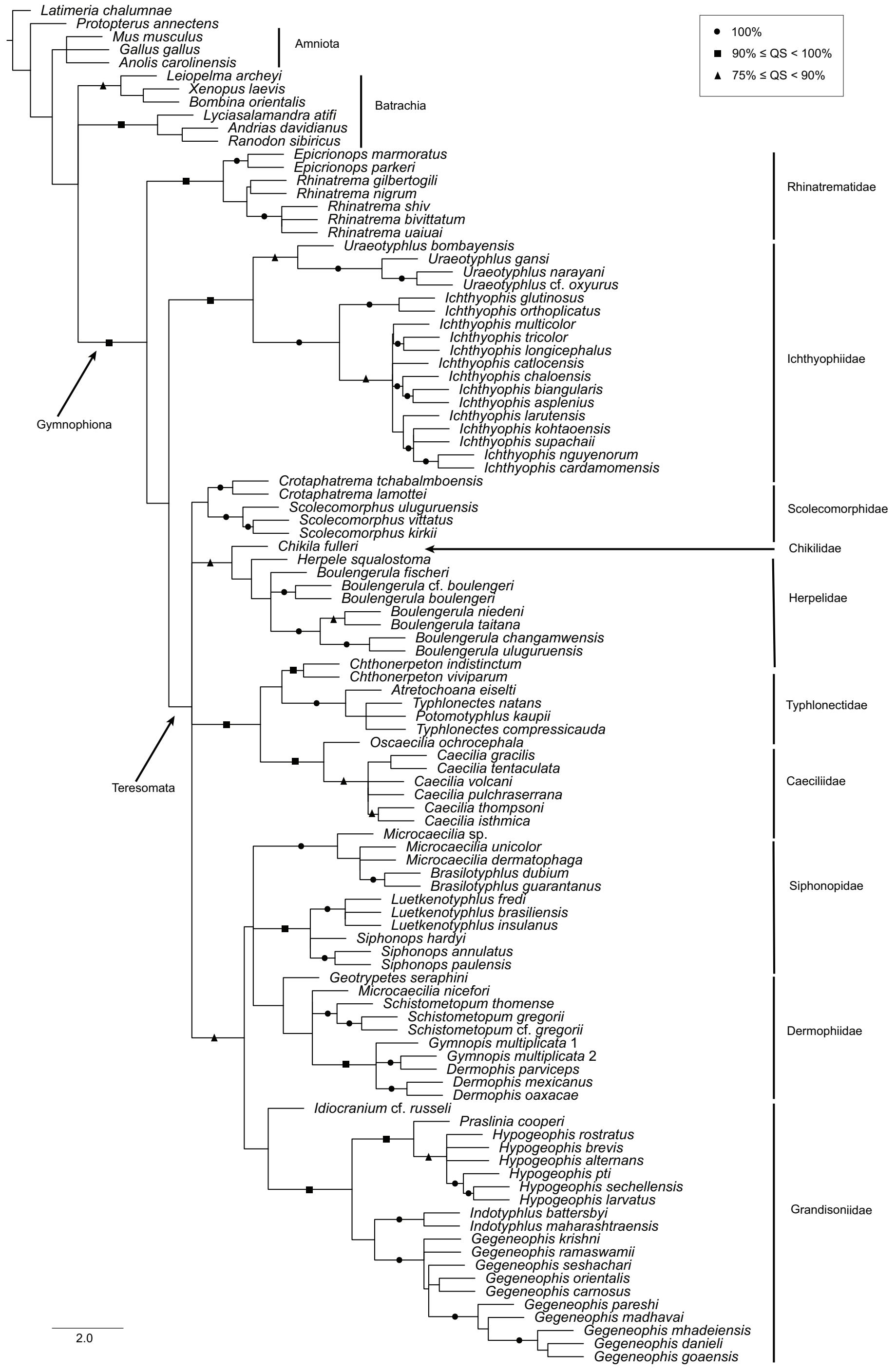

Figure S10: Astral supertree of the caecilian gene trees inferred with RAxML without *H. montanus* data. All branches with QS below 50% collapsed. Circles denote branches with 100% QS, squares denote 90% $\geq$ QS $<$ 100% and triangles 75% $\geq$ QS $<$ 90%. Scale bar represents coalescent units.

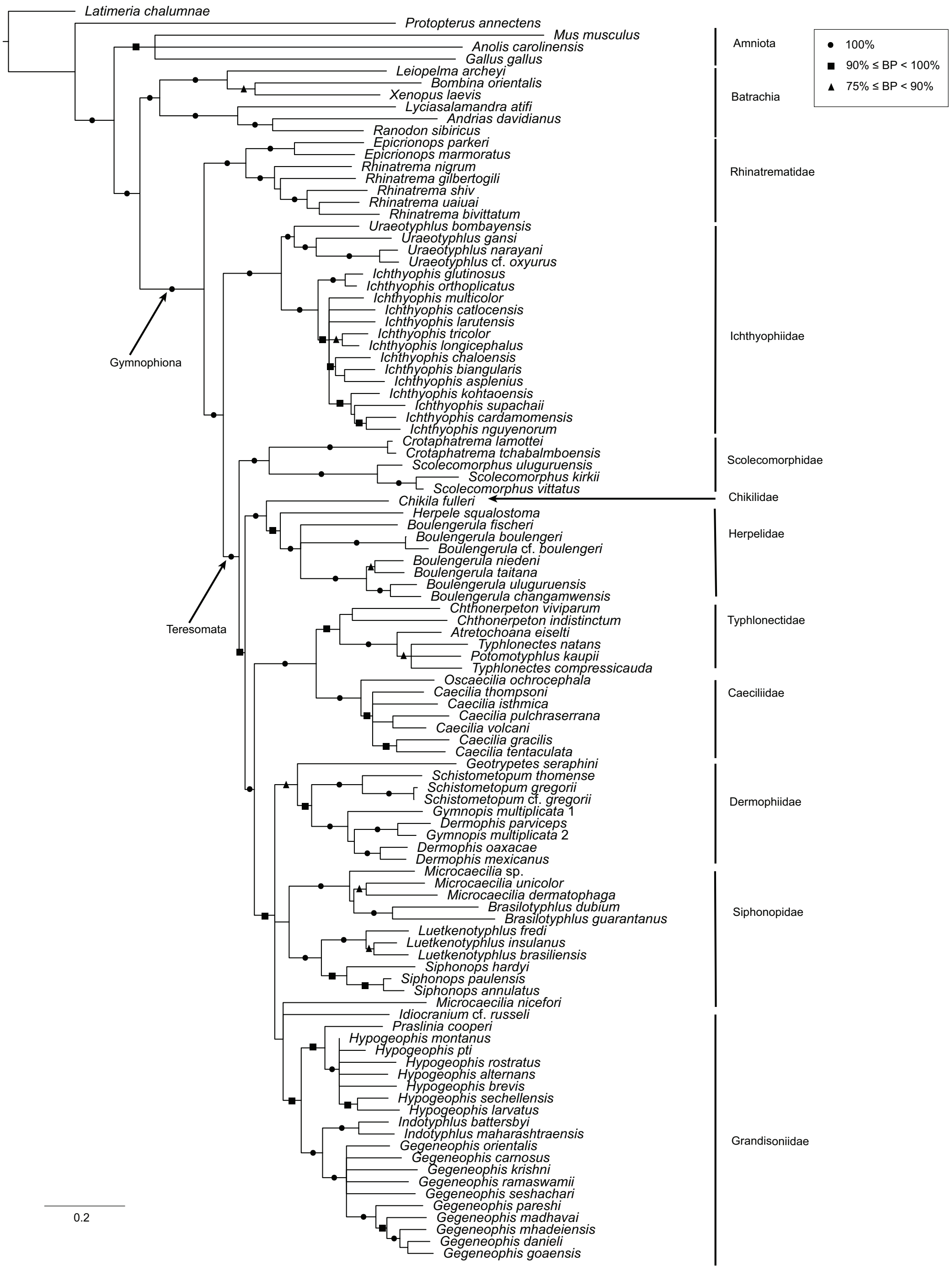

Figure S11: Best RAxML tree inferred from the concatenated caecilian matrix missing *H. montanus* data for the 16S rRNA locus. All branches with bootstrap proportion (BP) below 50% collapsed. Circles denote branches with 100% BP, squares denote 90% $\geq$ BP $<$ 100% and triangles 75% $\geq$ BP $<$ 90%. Scale bar represents estimated substitutions per site.

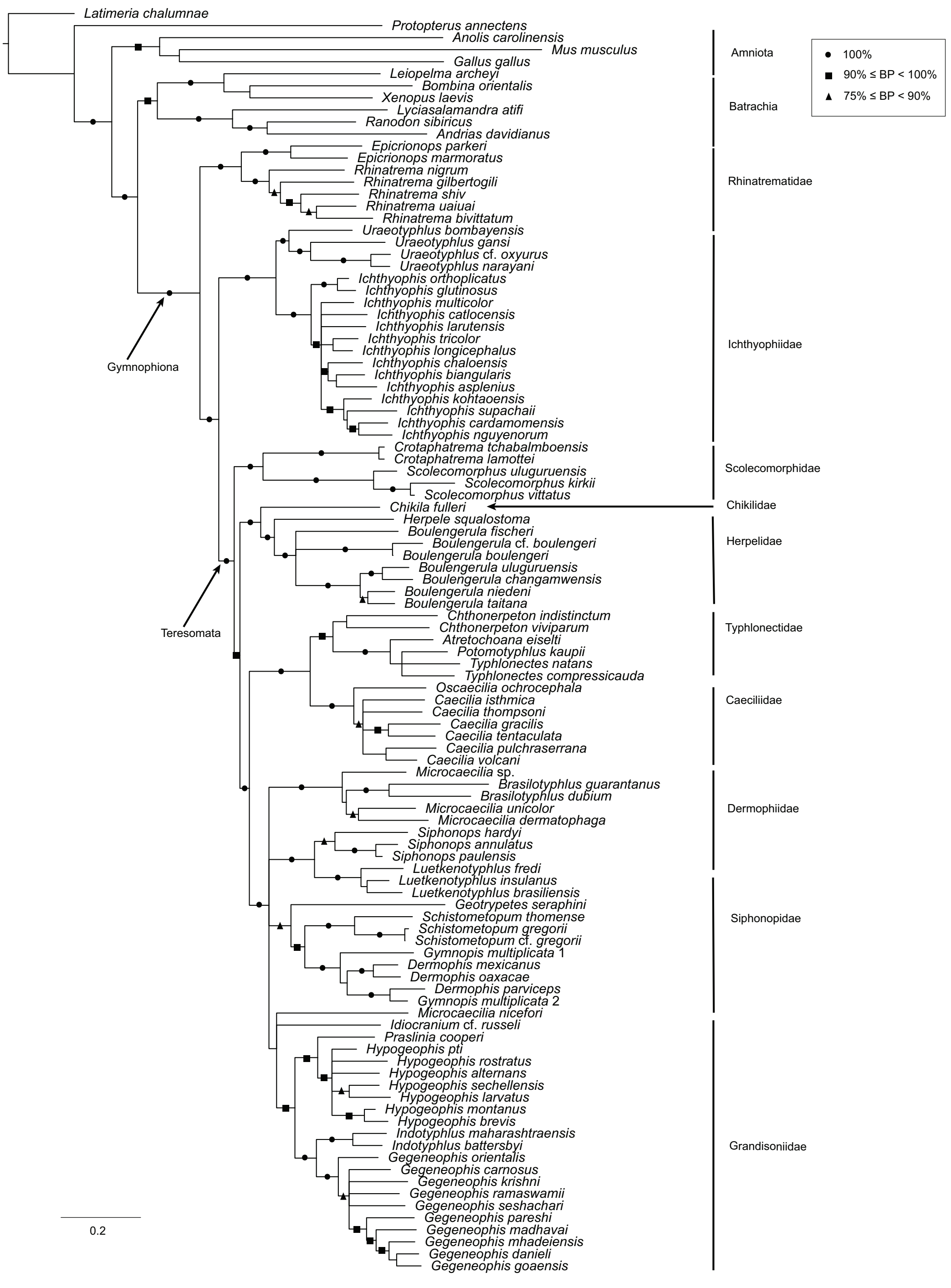

Figure S12: Best RAxML tree inferred from the concatenated caecilian matrix with *H. montanus* data for the 16S rRNA locus. All branches with BP below 50% collapsed. Circles denote branches with 100% BP, squares denote 90% $\geq$ BP $<$ 100% and triangles 75% $\geq$ BP $<$ 90%. Scale bar represents estimated substitutions per site.

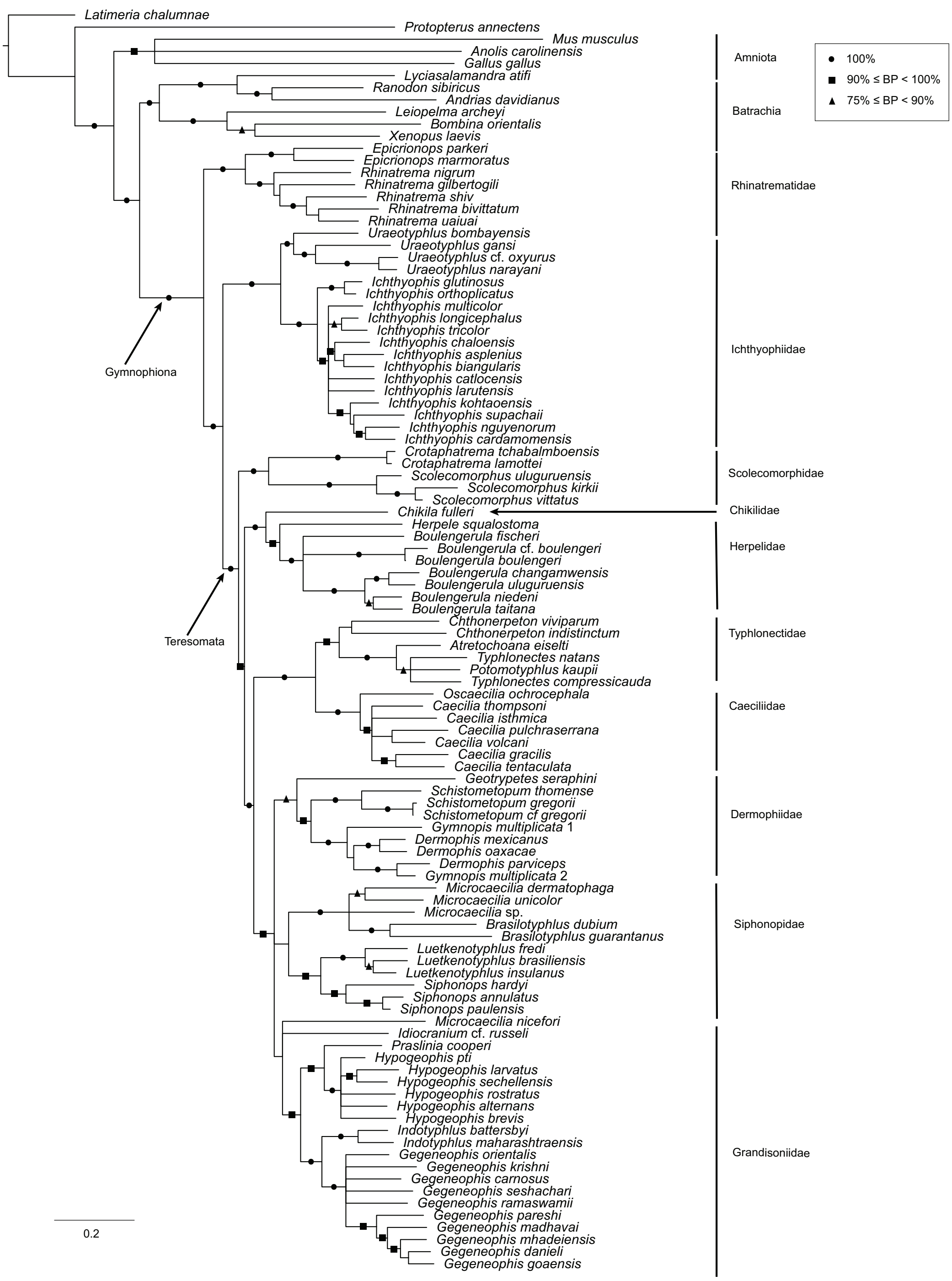

Figure S13: Best RAxML tree inferred from the concatenated caecilian matrix with no *H. montanus* data. All branches with BP below 50% collapsed. Circles denote branches with 100% BP, squares denote $90\% \geq \text{BP} < 100\%$ and triangles $75\% \geq \text{BP} < 90\%$. Scale bar represents estimated substitutions per site.

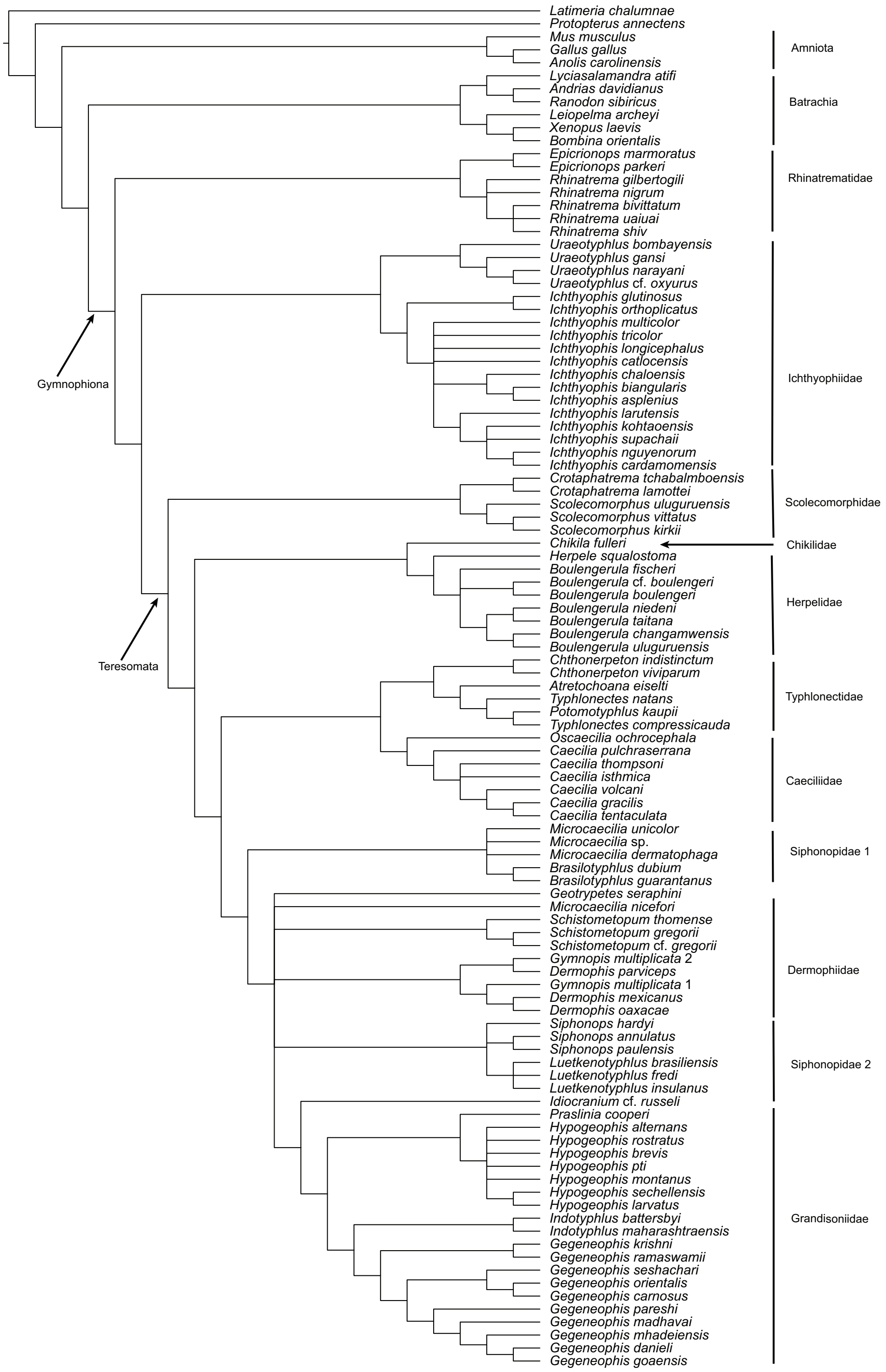

Figure S14: SC of the MRP tree inferred from the RAxML gene trees without 16S data for *H. montanus*.

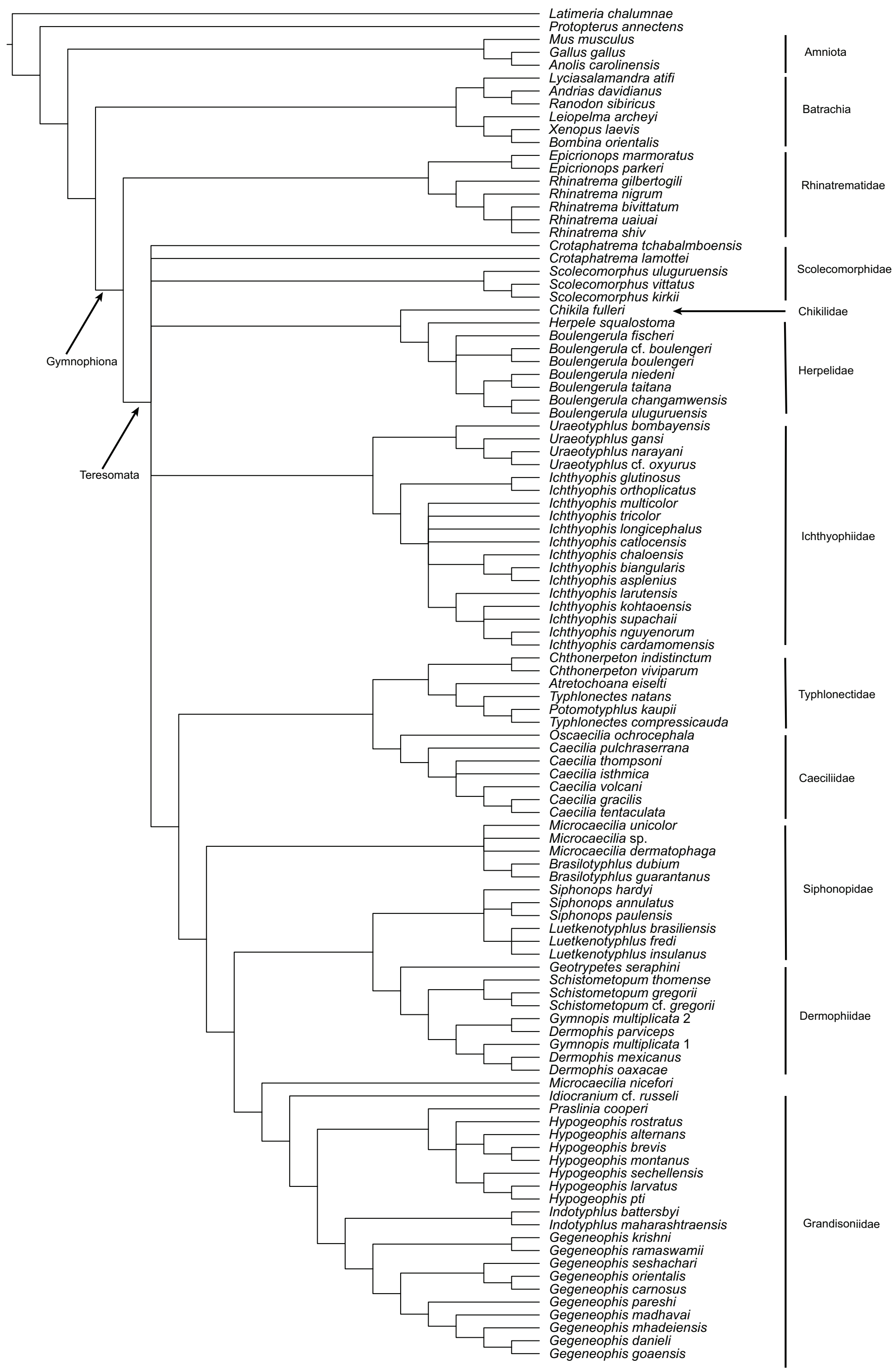

Figure S15: SC of the MRP tree inferred from the RAxML gene trees with 16S data for *H. montanus*.

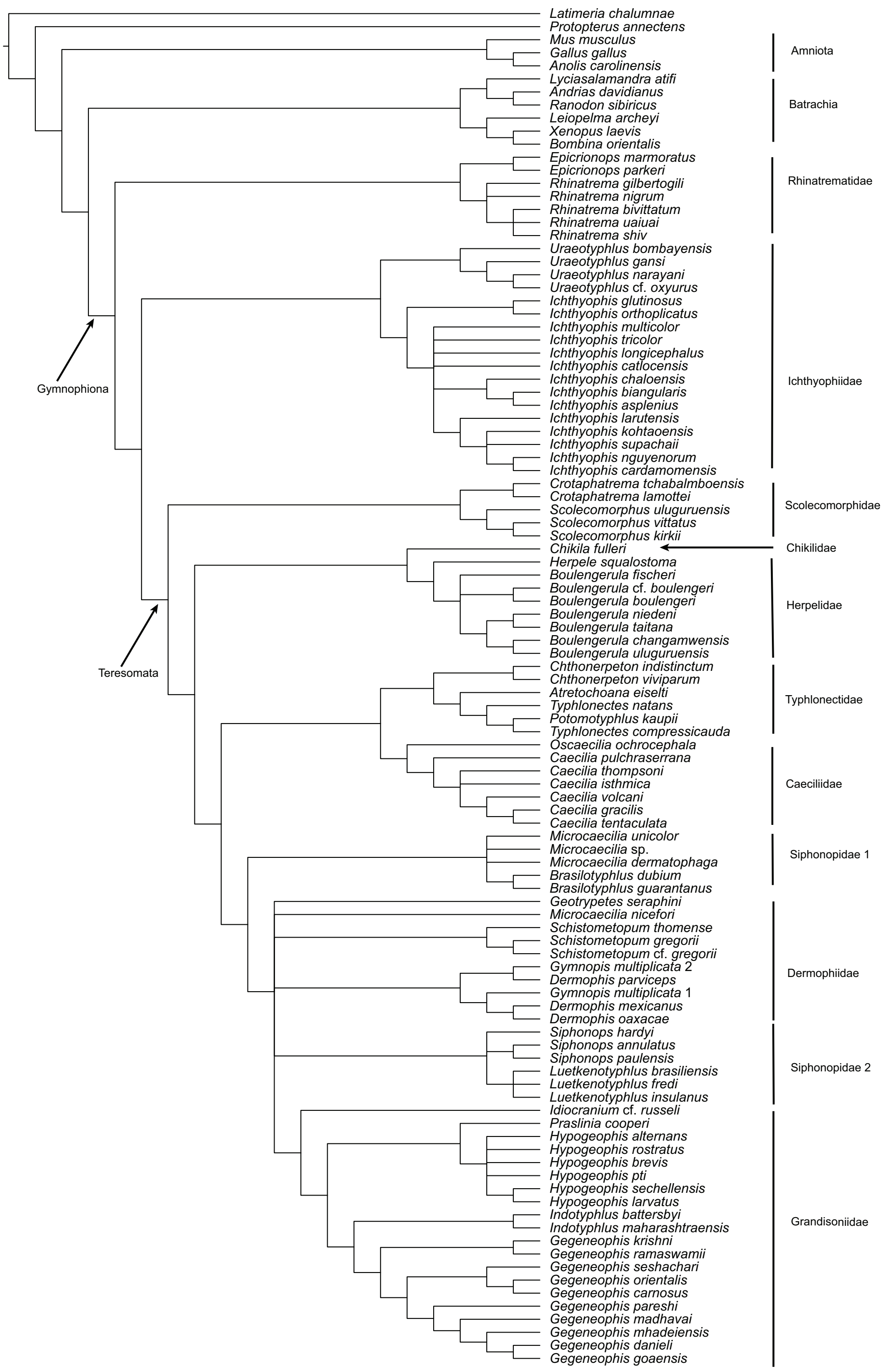

Figure S16: SC of the MRP tree inferred from the RAxML gene trees without *H. montanus* data.

4 References

Baum, B. R. and M. A. Ragan. 2004. The MRP method. Pages 17–34 *in* Phylogenetic Supertrees: Combining Information to Reveal the Tree of Life (O. R. P. Bininda-Emonds, ed.). Springer, Dordrecht.

Benson, D. A., I. Karsch-Mizrachi, D. J. Lipman, J. Ostell, and E. W. Sayers. 2008. GenBank. Nucleic Acids Res. 37:D26–D31.

Colless, D. H. 1980. Congruence between morphometric and allozyme data for *M*enidia species: a reappraisal. Syst. Zool. 29:288–299.

Dobrin, B. H., D. J. Zwickl, and M. J. Sanderson. 2018. The prevalence of terraced treescapes in analyses of phylogenetic data sets. BMC Evol. Biol. 18:46.

Margush, T. and F. McMorris. 1981. Consensus n-trees. Bull. Math. Biol. 43:239–244.

Meredith, R. W., J. E. Janečka, J. Gatesy, O. A. Ryder, C. A. Fisher, E. C. Teeling, A. Goodbla, E. Eizirik, T. L. L. Simão, T. Stadler, D. L. Rabosky, R. L. Hoenycutt, J. J. Flynn, C. M.Ingram, C. Steiner, T. L. Williams, T. J. Robinson, A. Burk-Herrick, M. Westerman, N. A. Ayoub, M. S. Springer and W. J. Murphy. 2011. Impacts of the Cretaceous Terrestrial Revolution and KPg extinction on mammal diversification. Science 334:521–524.

Rabosky, D. L., S. C. Donnellan, M. Grundler, and I. J. Lovette. 2014. Analysis and visualization of complex macroevolutionary dynamics: an example from Australian scincid lizards. Syst. Biol. 63:610–627.

Robinson, D. F. and L. R. Foulds. 1981. Comparison of phylogenetic trees. Math. Biosci. 53:131–147.

Ronquist, F., M. Teslenko, P. Van Der Mark, D. L. Ayres, A. Darling, S. Höhna, B. Larget, L. Liu, M. A. Suchard, and J. P. Huelsenbeck. 2012. MrBayes 3.2: efficient Bayesian phylogenetic inference and model choice across a large model space. Syst. Biol. 61:539–542.

Sanderson, M. J., M. M. McMahon, A. Stamatakis, D. J. Zwickl, and M. Steel. 2015. Impacts of terraces on phylogenetic inference. Syst. Biol. 64:709–726.

Shannon, P., A. Markiel, O. Ozier, N. S. Baliga, J. T. Wang, D. Ramage, N. Amin, B. Schwikowski, and T. Ideker. 2003. Cytoscape: a software environment for integrated models of biomolecular interaction networks. Genome Res. 13:2498–2504.

Shi, J. J. and D. L. Rabosky. 2015. Speciation dynamics during the global radiation of extant bats. Evolution 69:1528–1545.

Siu-Ting, K., D. Pisani, C. J. Creevey, and M. Wilkinson. 2015. Concatabominations: identifying unstable taxa in morphological phylogenetics using a heuristic extension to safe taxonomic reduction. Syst. Biol. 64:137–143.

Springer, M. S., R. W. Meredith, J. Gatesy, C. A. Emerling, J. Park, D. L. Rabosky, T. Stadler, C. Steiner, O. Ryder, J. E. Janečka, C. A. Fisher and W. J. Murphy. 2012. Macroevolutionary dynamics and historical biogeography of primate diversification inferred from a species supermatrix. PloS One 7:e49521.

Stamatakis, A. 2014. RAxML version 8: a tool for phylogenetic analysis and post-analysis of large phylogenies. Bioinformatics 30:1312–1313.

Swofford, D. L. 2003. PAUP*: phylogenetic analysis using parsimony, version 4.0 a165. Sinauer Associates, Sunderland, Massachusetts.

Thorley, J. L., M. Wilkinson, and M. Charleston. 1998. The information content of consensus trees. Pages 91–98 *in* Advances in Data Science and Classification: Proceedings of the 6th Conference of the International Federation of Classification Societies (IFCS-98) Università "La Sapienza", Rome, 21–24 July, 1998 (A. Rizzi, M. Vichi and H.-H. Bock,eds.) Springer, Berlin-Heidelberg.

Tolley, K. A., T. M. Townsend, and M. Vences. 2013. Large-scale phylogeny of chameleons suggests African origins and Eocene diversification. Proc. R. Soc. B: Biol. Sci. 280:20130184.

Wilkinson, M. 1995. Coping with abundant missing entries in phylogenetic inference using parsimony. Syst. Biol. 44:501–514.

Zanne, A. E., D. C. Tank, W. K. Cornwell, J. M. Eastman, S. A. Smith, R. G. FitzJohn, D. J. McGlinn, B. C. O'Meara, A. T. Moles, P. B. Reich, et al. 2014. Three keys to the radiation of angiosperms into freezing environments. Nature 506:89–92.

Zhang, C., M. Rabiee, E. Sayyari, and S. Mirarab. 2018. Astral-III: polynomial time species tree reconstruction from partially resolved gene trees. BMC Bioinf. 19:153.

Zwickl, D. 2014. Terraphy: Various analyses and utilities related to phylogenetic terraces. https://github.com/zwickl/terraphy
